# Exploration the role of pro-inflammatory fibroblasts and related markers in periodontitis: combing with scRNA-seq and bulk-seq data

**DOI:** 10.3389/fimmu.2025.1537046

**Published:** 2025-04-30

**Authors:** Erli Wu, Feihan Gu, Qiangqiang Zhuo, Ziyang Gao, Yu Zhang, Jingjing Li, Xuan Yin, Weimin Bao, Xianqing Zhou, Feng Liang, Shouxiang Yang, Yuanyin Wang, Qingqing Wang, Wei Shao

**Affiliations:** ^1^ Key Lab. of Oral Diseases Research of Anhui Province, College & Hospital of Stomatology, Anhui Medical University, Hefei, China; ^2^ Department of Periodontology, Anhui Stomatology Hospital affiliated to Anhui Medical University, Hefei, China; ^3^ Department of Microbiology and Parasitology, Anhui Provincial Laboratory of Pathogen Biology, School of Basic Medical Sciences, Anhui Medical University, Hefei, Anhui, China

**Keywords:** pro-inflammatory, fibroblasts, periodontitis, machine learning, feature genes

## Abstract

**Background:**

Gingival fibroblasts (GFs), as a critical component of periodontal tissue, play a vital role in processes such as collagen synthesis, wound healing, and tissue repair, thereby maintaining the structural integrity of periodontal tissues. Interestingly, recent studies have revealed that GFs also contribute to the pathophysiology of periodontitis by promoting inflammatory responses. However, its specific molecular mechanism and clinical relevance are still not fully understood.

**Methods:**

To find pro-inflammatory gingival fibroblasts (PIGFs) in periodontitis, a comprehensive analysis of single-cell RNA sequencing (scRNA-seq) data from normal and periodontitis patients was conducted. Then, the role of this celltype in periodontitis was further explored by using cell communication. By merging bulk transcriptome data and employing multiple machine learning algorithms, potential feature genes with PIGFs were further screened, which were verified by qPCR and immunofluorescence staining. Lastly, a cell function test was used to examine the part these genes play in the pathogenesis of periodontitis.

**Results:**

Through single-cell sequencing analysis, we identified PIGFs which were closely related to the development of periodontitis. Cell communication analysis revealed the specific role of PIGFs in periodontitis. Differential gene analysis, WGCNA, and machine learning algorithms identified two genes (MME and TSPAN11) as potential therapeutic targets for periodontitis. Immune infiltration analysis demonstrated a significant correlation between these genes and the immune response. Functionally, down-regulation of MME and TSPAN11 promoted the proliferation and migration of GFs and significantly inhibited the release of inflammatory cytokines and chemokines.

**Conclusion:**

This study identified a subpopulation of GFs closely associated with the inflammatory response through scRNA-seq analysis. These cells may contribute to the progression of periodontitis by interacting with various immune and non-immune cell types. Notably, MME and TSPAN11 were identified as key genes associated with this specific GFs subpopulation that may drive disease progression by exacerbating the inflammatory response, suggesting their potential as therapeutic targets for periodontitis.

## 1 Introduction

Periodontitis is a prevalent inflammatory condition that results in the deterioration of the supportive structures surrounding the teeth, potentially leading to tooth loss and systemic inflammation (1). According to statistics, periodontal disease affects 20% to 50% of people globally. In adults, it is one of the main causes of tooth loss. Additionally, there was a 57.3% increase in the frequency of periodontal disease globally between 1990 and 2010 (2). Periodontitis is caused by dysbiota and dysregulation of immune homeostasis, and severe periodontitis represents a significant health and socio-economic burden due to its health effects and high treatment costs (3, 4). The objectives of prevention and treatment include managing bacterial biofilms, addressing risk factors, stopping disease progression, and restoring lost tooth support (5). Nevertheless, the pathogenic processes behind periodontitis are still not fully understood (6).

An essential component of periodontal tissue, gingival fibroblasts (GFs) are in charge of preserving the integrity and structure of the tissue. Additionally, they can also function as sentinel cells to modulate the immune system’s reaction to oral infections that invade gingival tissue and play a part in the etiology of periodontitis (7). GFs are a crucial “non-classical” element of the innate immune system that produce cytokines, chemokines, and other inflammatory mediators in response to signals associated with germs and injury (8). But like immune cells, when they become overactive, they can cause tissue damage and chronic inflammation by secreting proteolytic enzymes including matrix metalloproteinases (MMPs) and cathepsins, overrecruiting white blood cells, and stimulating the production of osteoclasts (7). Notably, recent studies have identified specific subpopulations of pro-inflammatory gingival fibroblasts (PIGFs) in periodontitis. Qian et al. (9) reported the presence of a CXCL13^+^ GFs subpopulation in chronic periodontitis, which contributes to inflammation and tissue destruction. Therefore, exploring the specific pathways and key genes associated with this subpopulation may provide valuable insights for developing targeted therapeutic strategies for periodontitis in the future.

Conventional transcriptome bulk sequencing can only retrieve the average quantity of gene expression for distinct cell types in a sample. Therefore, it is challenging to distinguish and define various cell states and types using conventional transcriptome bulk sequencing, which can also lose information on significant cell subtypes (10). Nevertheless, single-cell sequencing is a novel technique that makes it possible to evaluate gene expression at the single-cell level. This provides information about tissue and cell heterogeneity, which is very helpful in understanding how cells differ among various illnesses (11).

In our study, we performed scRNA-seq analysis to identify PIGFs, which were most associated with the onset of periodontitis. By using cell communication analysis, we were able to thoroughly examine the role of PIGFs in periodontitis tissues. Subsequently, we combined with WGCNA and machine learning methods using the bulk RNA-seq dataset to search potential signature genes associated with PIGFs that may be involved in periodontitis progression. We also examined the correlation of these genes with immune cells, immune checkpoints and HLA genes using ssGSEA. Finally, our study confirmed the expression of PIGFs-related feature genes in periodontitis by qPCR and Immunofluorescence staining, and further investigated the role of these genes by cell transfection. Overall, our research provided a preliminary evaluation of the potential function of PIGFs in periodontitis and identified key characteristic genes associated with PIGFs. These findings may enhance our understanding of the pathogenesis of periodontitis and offer potential therapeutic targets for its treatment.

## 2 Materials and methods

### 2.1 Data acquisition

The GEO database (https://www.ncbi.nlm.nih.gov/geo/) provided us with the scRNA-seq (GSE164241) and bulk RNA-seq (GSE16134 and GSE10334) data for analysis. GSE164241 was used as a single-cell dataset for scRNA-seq research, containing 8 individuals with periodontitis and 13 healthy subjects. In terms of bulk RNA-seq datasets, GSE16134 consisted of 310 samples, of which 69 healthy samples and 241 disease samples served as the training dataset, while GSE10334 consisted of 247 samples, of which 64 normal and 183 illness samples served as the validation dataset. For the scRNA-seq dataset, the periodontal group inclusion criteria were defined as moderate to severe periodontitis, characterized by a probing depth (PD) >5 mm in more than four interproximal sites, along with clinical signs of inflammation such as erythema, edema, and bleeding on probing (BoP). For the bulk RNA-seq datasets, tissue samples were collected from patients with moderate to severe periodontitis, with “diseased” sites meeting the following criteria: BoP, interproximal PD≥3 mm, and concomitant attachment loss (AL)≥2 mm.

### 2.2 scRNA-seq data processing

The R “Seurat” software package (version 4.3.1) was utilized for the processing of scRNA-seq data (12). First, we utilized data quality techniques to eliminate cells with mitochondrial gene expression levels above 20% and gene expression levels below 200 or above 4,000 genes in order to ensure that the majority of cells were included in the data set used. Then, the “LogNormalize” function was used to normalize the data. After that, we used the “Harmony” package to remove batch effects between various data sets in accordance with the provided tutorial before doing principal component analysis (PCA) (13). We used the TSNE function to visualize the clustering cells. In order to identify preferentially expressed genes within clusters, we utilized the “FindAllMarkers” function in the “Seurat” package with logFC>0.25 and min.pct>0.25 as thresholds. Finally, we annotated each cell cluster using known cell type marker genes. The subcluster analysis is the same as the above steps.

### 2.3 Identification of DEGs

For bulk RNA-seq analysis, we used the “limma” package to compare differences in the expression of characteristic genes between samples with and without periodontitis. LogFC > 0.5 and adjusted p values <0.05 were used as DEG screening criteria. Heatmaps and volcano maps were used to visualize results.

### 2.4 The weighted gene co-expression network analysis network construction and module identification

The co-expression network was constructed using the “WGCNA” software. The following networks were made possible by using a soft threshold to guarantee the network’s scalability. Topological overlap matrix (TOM)-based hierarchical clustering was used to generate gene modules with strong linkages after gene modules were found using hierarchical clustering trees. Disease-module correlations were evaluated using Pearson’s correlation coefficient, and genes present in the module that had the highest disease correlation coefficient were selected for subsequent analysis.

### 2.5 Acquisition and pathway enrichment of hub genes

Through the intersection of transcriptome DEGs, PIGFs differential genes and WGCNA core module genes, PIGFs-related hub genes were obtained. Pathway enrichment of GO and KEGG was performed through the “clusterProfiler” package, and the 10 most significant important signaling pathways were selected.

### 2.6 Identification of diagnostic markers through machine learning

To choose the most suitable feature genes, we employed two machine learning techniques, the random forest (RF) algorithm and least absolute shrinkage and selection operator (LASSO) regression, to forecast illness stages and pinpoint crucial diagnostic factors. For RF analysis, we determined gene relevance and chose the top 5 genes of gene importance using the R “randomForest” package. Meanwhile, we performed LASSO regression analysis for lambda min values using the R “glmnet” package. The core genes of PIGFs were determined to be the intersection genes that were acquired using the two machine learning techniques.

### 2.7 Expression levels and diagnostic utility of potential biomarkers

The study compared and examined the expression levels of feature genes using the “limma” and “ggpubr” R packages. Boxplots were utilized to present the findings. Receiver Operating Characteristic (ROC) curve analysis was performed simultaneously for each core gene using the “pROC” software, and the area under the curve (AUC) was calculated (14).

### 2.8 ssGSEA and GSEA (gene set enrichment analysis) for the model gene

The ssGSEA algorithm was utilized by R package “GSVA” to comprehensively evaluate the Hallmark gene set in the study. In GSEA, genes were defined functionally in order to clarify their biological significance. The samples were separated into two categories based on the model’s median gene expression value, and differential genes between two groups were retrieved for examination. The relationship between linked gene sets and hub genes was examined using Spearman analysis.

### 2.9 Immune correlation analysis

The percentage of 22 immune cells in samples with periodontitis and healthy samples was evaluated using the CIBERSORT technique. Afterwards, the relationship between pivot genes and immune infiltrating cells was examined using the Spearman coefficient. Additionally, we examined the relationships between these genes and chemokine-related and HLA genes in order to look into the immunological role of hub genes in the onset and progression of periodontitis. It was deemed statistically significant when P ≤ 0.05.

### 2.10 Collection of periodontal tissue specimens

Samples of gingival tissue, 10 in good health and 10 with periodontitis, were taken from patients at the Anhui Medical University’s Affiliated Stomatology Hospital in Hefei, Anhui Province. The Anhui Medical University Affiliated Stomatology Hospital’s Ethics Committee reviewed and approved the study (Ethics number: 2021006). Every participant provided written informed permission.

### 2.11 RNA extraction and quantitative real-time PCR

The TRIzol reagent (Thermo Fisher Scientific, USA) was utilized to extract the gingival tissues’ total RNA, which was then reverse-transcribed into cDNA. SYBR premixed solution Ex Taq (TaKaRa) was used for qRT-PCR. Formula^2−ΔCT^ was used to compute the expression value using the comparative CT method, and each gene’s expression levels were normalized to GAPDH. The primer sequences for the genes used in this investigation were reported in **Supplementary Table 1**.

### 2.12 Immunofluorescence assay

The collected gingival tissue was fixed, embedded, sliced and then antigen was extracted. After applying sheep serum to the slide, the primary antibody was incubated overnight. We used a secondary antibody that recognized the primary antibody to stain the sections for an hour at room temperature, and then we used DAPI to stain them for three minutes. The stained slices were photographed using an imaging Zeiss 800 laser scanning confocal microscope.

### 2.13 Cell culture

Gingival tissues were obtained as described above. Use PBS gingival tissue and then cut the gingival tissue into small pieces, which was used in Dulbecco’s modified Eagle medium (α-MEM; Gibco, USA) and 10% fetal bovine serum (OriCell, China) at 37°C and 5% CO2. Seven days later, primary cells began to proliferate outside the tissue block. When the cells reach 80% confluent, they are passed and cultured. We used cells from passages 4-8. GFs were cultured with *Porphyromonas gingivalis* lipopolysaccharide (Pg-LPS, InvivoGen, France) for 6 hours to induce periodontitis *in vitro*.

### 2.14 Cell transfection

MME and TSPAN11 were targeted using short interfering RNA (siRNA) and negative control (NC) oligonucleotides (**Supplementary Table 2**). To conduct the cell culture experiment, GFs were inoculated in a 6-well plate. Liposome 2000 (Invitrogen, USA) was applied to the cells at a final concentration of 25 nM together with siRNA or control (General, Anhui, China) in accordance with the manufacturer’s instructions.

### 2.15 Proliferation assay with cell counting kit 8

Each 96-well plate was inoculated with 4,000 cells, and after transfection 0, 24, 48, 72, and 96 hours, 10 μl of CCK-8 reagent was added to the culture medium. After an hour of incubation at 37°C, the cells’ absorbance was measured with a microplate reader at 450 nm.

### 2.16 Wound healing test

After transferring transfected GFs to a 6-well plate, 80% fusion was achieved. After scraping the single cell layer with the tip of a 100 μl pipette and washing the cells three times with PBS to get rid of cell debris, the cells were then given fresh media that was free of serum. After scraping for 0 and 24 hours, representative pictures of cell migration were taken in three distinct high-magnification fields. Estimate the scratch’s width using the ImageJ program.

### 2.17 Statistical analysis

GraphPad Prism 8.0 and R software (version 4.3.1) were used for data processing and analysis. The data was expressed using the mean ± standard deviation. The unpaired T-test was the preferred statistical analysis approach, with a significance threshold of P-value <0.05.

## 3 Results

### 3.1 Identification of PIGFs by scRNA-seq analysis

**Figure 1:**
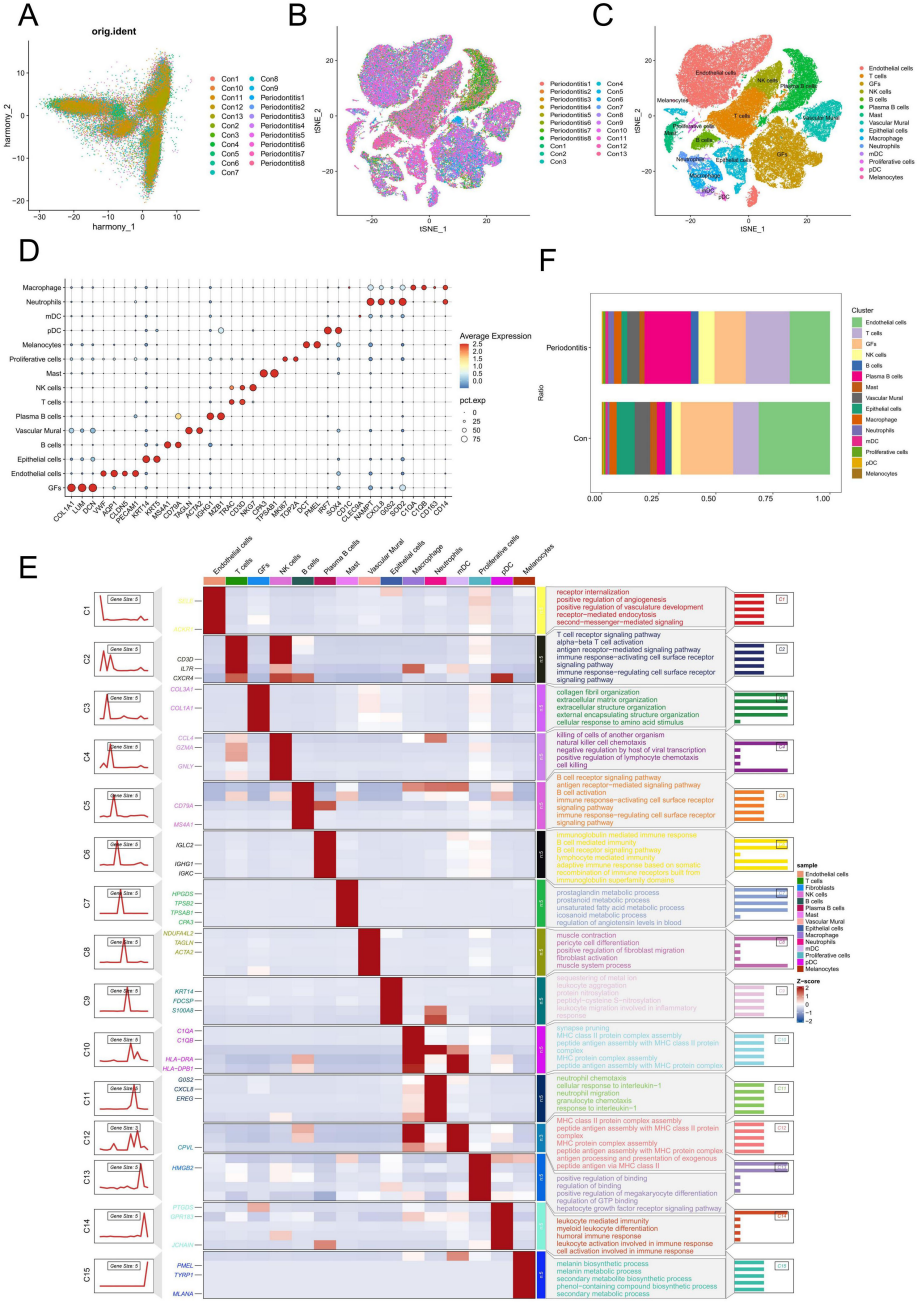
Single-cell analysis of cell proportion of periodontitis. **(A)** PCA plot showing the elimination of batch effects between different samples. **(B)** Cells on the t-SNE plot of all 21 samples were colored as originating from normal and periodontitis patients. **(C)** The t-SNE plot visualizes the distribution of 15 cell types in gingival samples. **(D)** Bubble maps were used to display surface-annotated genes for various cell types. **(E)** Heatmap showing representative differentially expressed genes between each celltype. The left panel depicts the dynamic expression patterns of representative DEGs across each cell type. The right panel presents the corresponding biological functions and pathways associated with each cell type, as identified through GO and KEGG analysis. **(F)** Cell proportions of 15 cell types originating from normal and periodontitis samples. GFs, Gingival fibroblasts; DEGs, differentially expressed genes.

We used the “Seurat” program in R to examine the scRNA-seq data from GSE164241. Following batch correction using PCA, no significant batch effects were observed (**Figure 1A**). Subsequently, a total of 15 distinct cell types were identified from 21 gingival samples, including T cells, NK cells, endothelial cells, GFs, plasma B cells, vascular murals, epithelial cells, neutrophils, macrophages, myeloid dendritic cells (mDCs), plasmacytoid dendritic cells (pDCs), B cells, Masts, melanocytes and proliferative cells (**Figures 1B, C**). The surface markers used for annotating each cell type are shown in **Figure 1D**. **Figure 1E** illustrated the genes and biological functions that were specifically and highly expressed within each of the 15 major cell clusters. The percentage of each cell type in the control and periodontitis samples was displayed in **Figure 1F**. Notably, the percentage of GFs was lower in periodontitis samples compared to the normal group, suggesting their potential involvement in the pathogenesis of periodontitis.

**Figure 2:**
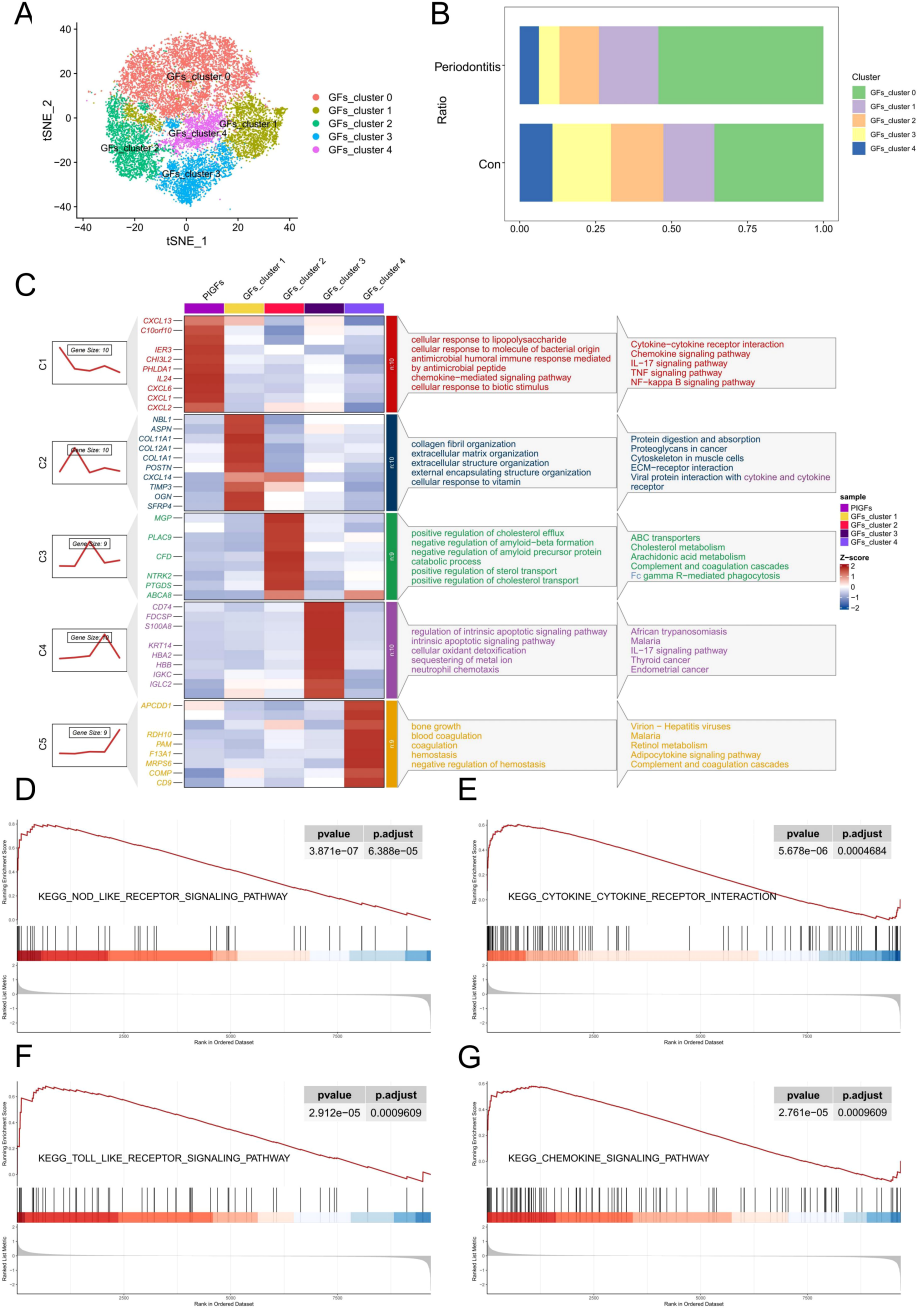
scRNA-seq analysis reveals heterogeneity of PIGFs subtypes in periodontitis samples. **(A)** Subclustering of GFs in normal and periodontitis samples further identified 5 distinct subtypes. **(B)** Cell proportions of GFs subclusters in the gingival tissues of normal and periodontitis patients. **(C)** Heatmap showing representative differentially expressed genes in each GFs subpopulation. The C1-C5 clusters represent the gene expression patterns of these different subpopulations as depicted by the gene trend plots on the left. The enriched gene ontology (GO) biological processes associated with each GFs subpopulation are shown on the right. **(D-G)** GSEA enrichment plots for representative signaling pathways upregulated in PIGFs compared to other GFs. GFs, Gingival fibroblasts; PIGFs, Pro-inflammatory gingival fibroblasts.

We re-clustered the GFs according to the above steps and identified a total of 5 GFs subclusters (**Figure 2A**). Interestingly, we discovered that, in comparison to the normal group, periodontitis samples had a much higher proportion of GFs cluster 0 (**Figure 2B**). Furthermore, some inflammatory cytokines and chemokines are highly expressed in it, such as CXCL1, CXCL2, CXCL6, CXCL13 and IL-24. Afterwards, we conducted functional and pathway enrichment analyses on the cluster, revealing significant enrichment of multiple inflammation-related functions and pathways, including cellular response to lipopolysaccharide (LPS) and bacterial-derived molecules, as well as the chemokine, NF-κB, and TNF signaling pathways (**Figure 2C**). Based on these findings, we labeled the cluster as pro-inflammatory gingival fibroblasts (PIGFs). Furthermore, GSEA was performed to explore the biological pathways associated with PIGFs. The results demonstrated that PIGFs were linked to several inflammatory signaling pathways, including cytokine-cytokine receptor interaction, NOD-like receptor signaling pathway, toll-like receptor signaling pathway and chemokine signaling pathway, all of which were strongly associated with the pathophysiology of periodontitis (**Figures 2D-G**).

### 3.2 Cell-cell communications analyses in PIGFs

**Figure 3:**
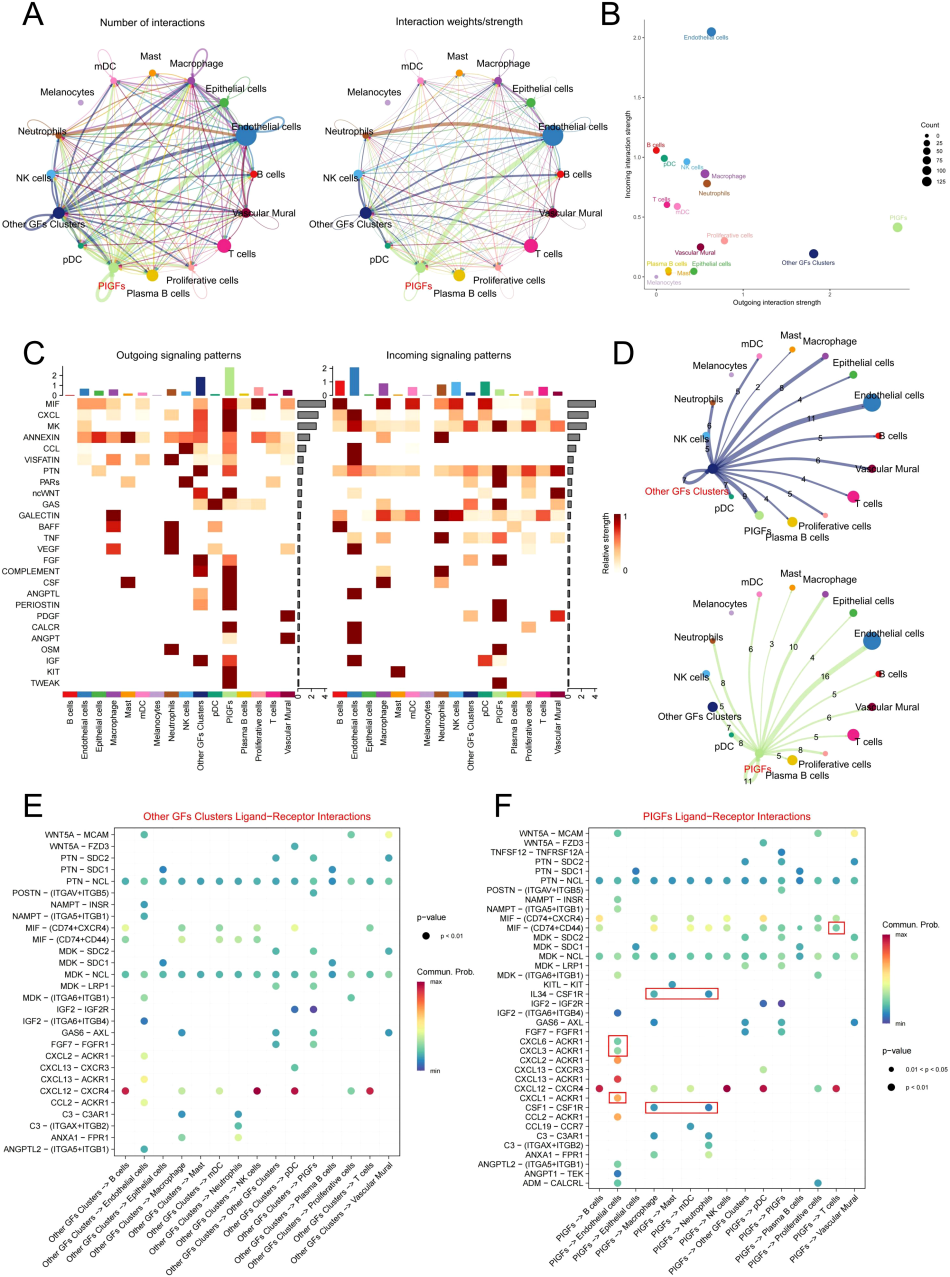
Cell communication analysis in GFs subpopulations. **(A)** Circle plots depict the number and strength of ligand-receptor interactions between pairs of cell populations. **(B)** A scatter plot reveals the variations in incoming and outgoing interaction strengths across all cell types. **(C)** The outgoing and incoming signaling patterns of GFs and other cell populations. **(D)** Circle plots depict the number and intensity of interactions between other GFs and PIGFs and other cells, respectively. The edges indicated the strength of the interactions, with thicker edges indicating stronger interactions. Numbers on the edges indicated the number of communication signals between the two cell types. **(E, F)** Bubble plot showing the significant ligand-receptor pairs between other GFs and PIGFs and other cells, respectively. The size of each dot represents the significance level (p-value), with larger dots indicating greater statistical significance. The color gradient reflects the strength of cellular communication, where redder hues denote stronger interactions. GFs, Gingival fibroblasts; PIGFs, Pro-inflammatory gingival fibroblasts.

By employing the “CellChat” algorithm, we investigated cell-cell communication and elucidated the role of PIGFs in disease pathophysiology. The results demonstrated that PIGFs engaged in extensive cellular interactions within the periodontal microenvironment (**Figure 3A**). Furthermore, PIGFs exhibited a broader capacity for both signal reception and transmission compared to other GFs (**Figures 3B, C**). **Figure 3D** presented cell interaction networks for PIGFs and other GFs, respectively. Notably, PIGFs exhibited stronger interactions with both immune and non-immune cell populations compared to other GFs. To further delineate these interactions, we analyzed and compared the specific ligand-receptor pairs associated with PIGFs. The analysis revealed that, in comparison to other GFs, PIGFs specifically interacted with neutrophils and macrophages via CSF1-CSFR1 and IL-34-CSFR1. Additionally, PIGFs engaged with T cells through the MIF-(CD74+CD44) signaling pathway (**Figures 3E, F**). These specific signaling pathways suggested that PIGFs might have enhanced immune cell recruitment, thereby exacerbating inflammatory responses and contributing to periodontal tissue damage. Interestingly, PIGFs were observed to interact with endothelial cells via CXCL1/CXCL3/CXCL6-ACKR1. Previous studies have suggested that endothelial cells expressing ACKR1 can be activated by pro-inflammatory chemokines, leading to increased local vascular permeability. This, in turn, facilitates the infiltration of immune cells into inflamed tissues, potentially amplifying the inflammatory response and contributing to tissue destruction (15). These findings suggested that PIGFs may play a role in enhancing vascular permeability in endothelial cells, thereby exacerbating immune responses and aggravating tissue damage.

### 3.3 Identification of differentially expressed genes

**Figure 4:**
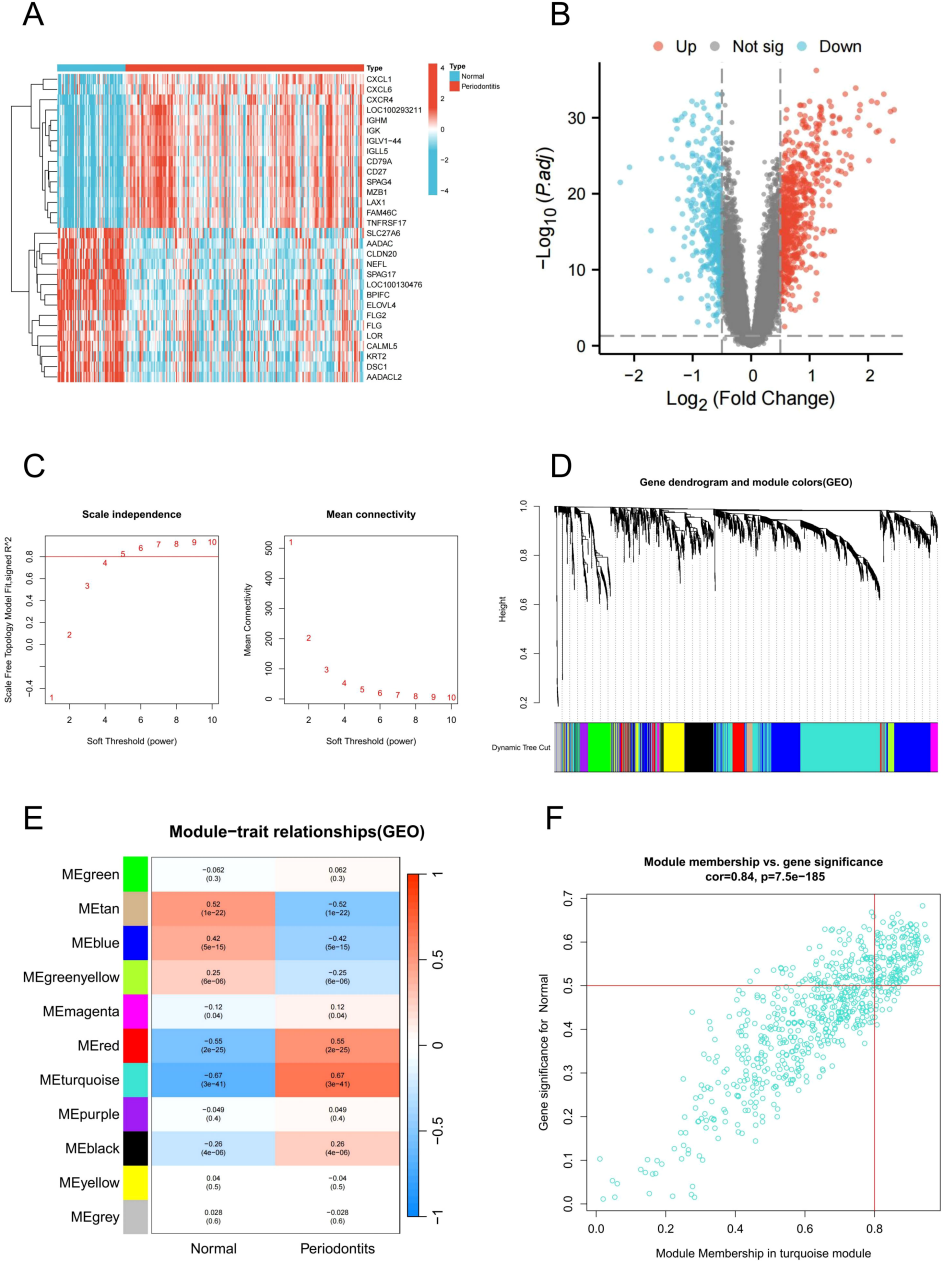
DEGs and WGCNA analysis in GSE16134. **(A)** Heatmap of DEGs. **(B)** Volcano plot of DEGs. **(C)** Soft threshold determination in GSE16134 **(D)** Clustering dendrogram of filtered genes based on a dissimilarity measure of topological overlap matrix. **(E)** Heatmap of module-trait correlations by WGCNA. **(F)** Scatter plot of gene significance for periodontitis and module membership in the MEturquoise module. DEGs, differentially expressed genes; WGCNA, Weighted gene co-expression network analysis.

The above findings suggest that PIGFs may play a role in the progression of periodontitis by exacerbating the inflammatory response. Therefore, identifying and targeting PIGFs-related genes may offer potential therapeutic strategies for periodontitis. To identify genes associated with PIGFs, we integrated bulk transcriptome data and analyzed DEGs between healthy and disease samples. We identified 1105 DEGs in GSE16134 using the “limma” package, of which 651 were up-regulated and 454 down-regulated. **Figure 4A** displayed the top 30 DEGs with the greatest differences, while **Figure 4B** displayed the differential genes volcano map.

### 3.4 WGCNA network construction and module identification

Subsequently, we performed WGCNA to identify core modules and genes associated with the pathogenesis of periodontitis. A robust sample clustering was achieved by checking for outliers and selecting an appropriate cutting threshold. To construct a scale-free network, a power of β = 5 was chosen (**Figure 4C**). The co-expression network that was constructed comprised of 11 modules. The modules most relevant to the disease were obtained by calculating their correlation coefficients (r) and P-values. With the highest positive association (r = 0.67), the turquoise module contained 690 genes that were used in further research (**Figures 4D, E**). In addition, scatter plots of gene significance in the module in relation to module membership indicated that these genes were suitable for gene mining (**Figure 4F**).

### 3.5 Acquisition and pathway enrichment of hub genes

**Figure 5:**
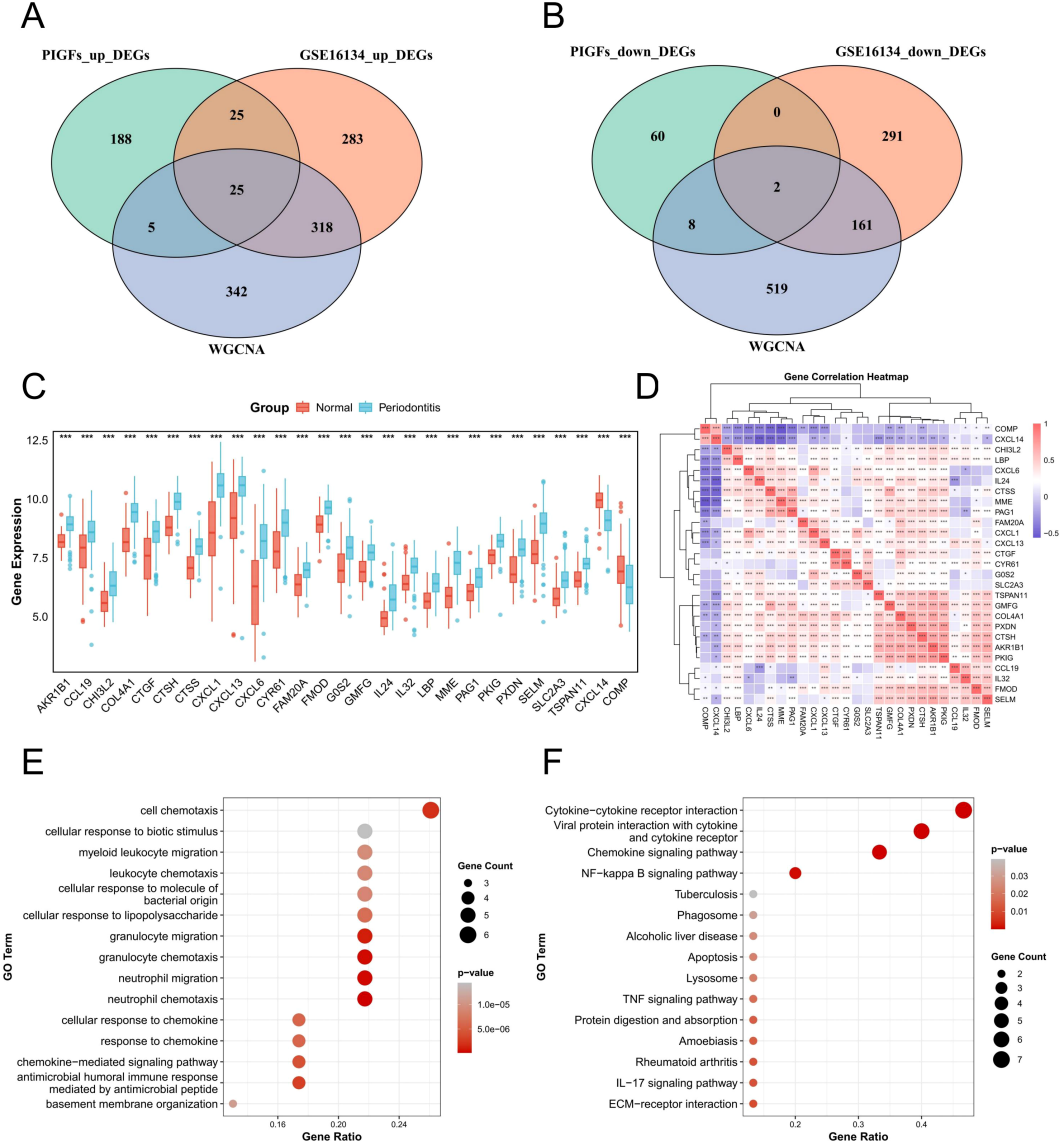
Expression analysis and functional enrichment of potential feature genes. **(A)** Venn diagram of the intersection of differentially upregulated genes in periodontitis, differentially upregulated genes in single-cell periodontitis PIGFs and WGCNA core module genes. **(B)** Venn diagram of the intersection of differential genes down-regulated in periodontitis, differential genes down-regulated in single-cell periodontitis osteoblasts and WGCNA core module genes. **(C)** Expression analysis of 27 potential feature genes between normal and periodontitis samples. **(D)** Correlation analysis between potential feature genes. **(E)** Enrichment analysis of potential feature genes using GO. **(F)** Enrichment analysis of potential feature genes using the KEGG. *p < 0.05, **p < 0.01, ***p < 0.001. DEGs, differentially expressed genes; WGCNA, Weighted gene co-expression network analysis.

In the scRNA-seq analysis, a total of 243 up-regulated and 70 down-regulated genes in PIGFs were obtained compared to other GFs subpopulations for logFC>0.25 and min.pct>0.25 threshold and **Supplementary Table 3** listed these genes. Subsequently, 25 commonly up-regulated genes and 2 down-regulated genes were obtained through the intersection of periodontitis differential genes, PIGFs differential genes and WGCNA core module genes (**Figures 5A, B**). The expression levels of these 27 potential signature genes in GSE16134 were shown using boxplots (**Figure 5C**). The results of our analysis of correlations between these unique genes indicated that these 27 genes may be closely related to each other (**Figure 5D**). Potential feature genes in GO enrichment analysis were shown to be enriched in cellular response to cell chemotaxis, LPS and granulocyte migration (**Figure 5E**). According to the KEGG enrichment analysis, these genes showed a substantial enrichment in pathways linked to several inflammatory signaling pathways, including NF-κB signaling pathway, chemokine signaling pathway, cytokine-cytokine receptor interaction, TNF signaling pathway and IL-17 signaling pathway (**Figure 5F**).

### 3.6 Identification the best characteristic genes of periodontitis by machine learning

**Figure 6:**
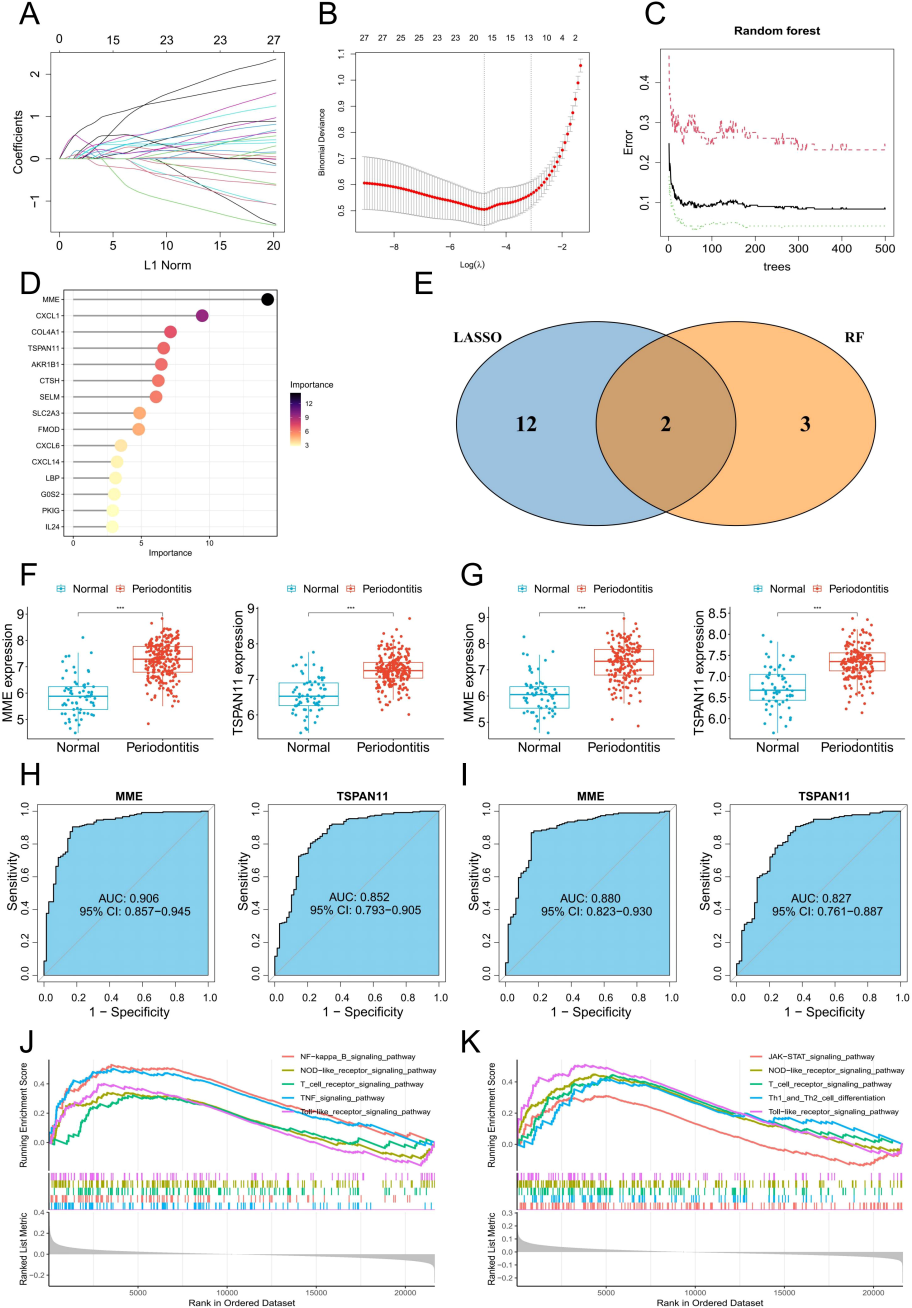
Machine learning identifies optimal feature genes of periodontitis. **(A)** Coefficient changes of the selected features using the lasso algorithm. **(B)** Lasso algorithm for selection features. **(C)** The impact of the number of decision trees on the error rate was examined. **(D)** The relative importance of potential feature genes was calculated in random forest (Top 5 genes’ importance > 2). **(E)** Venn diagram showing the overlap between the three algorithms. **(F)** MME and TSPAN11 mRNA expression in the training group. **(G)** MME and TSPAN11 mRNA expression in the testing group. **(H)** ROC curves of MME and TSPAN11 in the training group. **(I)** ROC curves of MME and TSPAN11 in the testing group. **(J, K)** GSEA identifies signaling pathways involved in MME and TSPAN11. ***p < 0.001.

To explore potential feature genes linked to PIGFs in periodontitis, we chose the machine learning techniques LASSO and RF for further analysis. 14 genes were produced by using the 27 genes mentioned above as inputs for LASSO regression (**Figures 6A, B**). We employed the RF algorithm to obtain 5 genes (**Figures 6C, D**). Lastly, we utilized the intersections of these gene sets to determine that MME and TSPAN11 were the most relevant characteristic genes linked to PIGFs (**Figure 6E**). **Supplementary Figures 1A, B** showed that these genes were primarily highly expressed in PIGFs, suggesting a close association with them. To validate the expression patterns of these key genes, we evaluated them using the training set GSE16134 and the validation set GSE10334. Consistent with the validation set’s results, our analysis showed that patients with periodontitis had increased expression levels of MME and TSPAN11 (**Figures 6F, G**). Additionally, we explored the potential relevance of these four genes in periodontitis using ROC curve analysis. The AUC values of the two genes, MME and TSPAN11, in the training set GSE16134 were 0.906 and 0.852, respectively (**Figure 6H**). Additionally, the AUC values for all five genes were greater than 0.8 in validation set GSE10334, highlighting a possible association with periodontitis (**Figure 6I**).

### 3.7 The ssGSEA and GSEA analysis

To further explore the biological functions, pathways, and correlations of the feature genes MME and TSPAN11, we conducted analyses using GSEA and ssGSEA algorithms. The GSEA results showed that MME and TSPAN11 were associated with a variety of inflammatory signaling pathways, including NF-κB signaling pathway, toll-like receptor signaling pathway, NOD-like receptor signaling pathway and T cell receptor signaling pathway (**Figures 6J, K**). Furthermore, we employed ssGSEA algorithms to evaluate 50 marker gene sets in periodontitis and found that multiple gene sets were significantly upregulated in periodontitis (**Supplementary Figure 2A**). We then investigated the association of two characteristic genes with these gene sets and found that MME and TSPAN11 were significantly associated with multiple gene sets (**Supplementary Figure 2B**).

### 3.8 Cell-cell communication analysis in MME and TSPAN11-associated GFs

**Figure 7:**
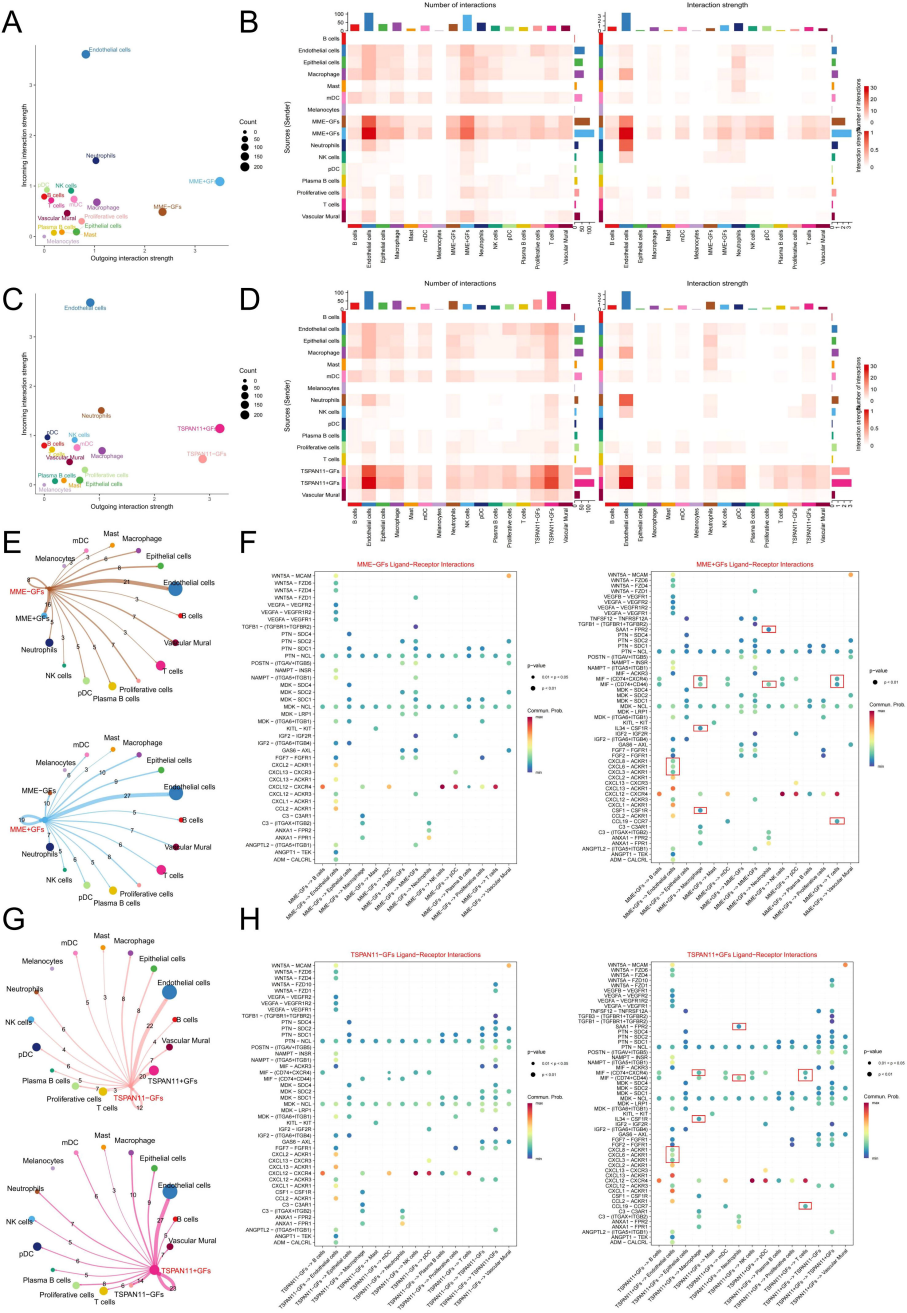
Cell-Cell Communication Analysis in MME and TSPAN11-Associated GFs. **(E)** A scatter plot showing the strength of input and output interactions between MME-associated GFs and other cell types. **(B)** A heatmap illustrating the strength and number of interactions between MME-associated GFs and other cell types. **(C)** A scatter plot showing the strength of input and output interactions between TSPAN11-associated GFs and other cell types. **(D)** A heatmap illustrating the strength and number of interactions between TSPAN11-associated GFs and other cell types. **(E)** A communication network associated with MME. The edges indicate the strength of interactions, with thicker edges representing stronger interactions. Numbers on the edges denote the number of communication signals between the two cell types. **(F)** A bubble plot showing the ligand-receptor pairs between MME^-^ and MME^+^ GF subpopulations and other cells. The size of each dot represents the significance level (p-value), with larger dots indicating greater statistical significance. The color gradient reflects the strength of cellular communication, where redder hues denote stronger interactions. **(G)** A communication network associated with TSPAN11. **(H)** A bubble plot showing the ligand-receptor pairs between TSPAN11^-^ and TSPAN11^+^ GF subpopulations and other cells. GFs, Gingival fibroblasts.

To further elucidate the functional role of hub genes in PIGFs, we employed scRNA-seq to classify PIGFs into positive (MME^+^ and TSPAN11^+^) and negative (MME^-^ and TSPAN11^-^) GFs based on hub gene expression profiles. Comparative analysis revealed that MME^+^ and TSPAN11^+^ GFs exhibited significantly enhanced intercellular communication capabilities, demonstrating both increased interaction intensity and number compared to their negative counterparts (**Figures 7A-D**). Detailed investigation of cellular interactions demonstrated that MME^+^ GFs, functioning as signal senders, established robust communication networks with multiple cell types, including neutrophils, T cells, macrophages, and endothelial cells (**Figure 7E**). The molecular mechanisms underlying these interactions were defined by specific ligand-receptor pathways, which were uniquely observed in MME^+^ GFs, acting on target cells through the following pathways: (1) macrophage interaction via GSF1-GSFR1, IL34-CSFR1, MIF-(CD74+CD44), and MIF-(CD74+CXCR4) pathways; (2) neutrophil regulation through MIF-(CD74+CD44) and SAA1-FPR2 pathways; (3) T cell modulation via CCL19-CCR7, MIF-(CD74+CD44), and MIF-(CD74+CXCR4) pathways; and (4) endothelial cell signaling through CXCL3/CXCL6/CXCL8-ACKR1 pathways (**Figure 7F**). Similarly, TSPAN11^+^ GFs displayed enhanced immunomodulatory properties compared to TSPAN11^-^ GFs (**Figure 7G**), establishing significant interactions with immune cells through distinct molecular pathways that were uniquely associated with the TSPAN11^+^ population: (1) macrophage regulation via the specific IL34-CSFR1 and MIF-(CD74+CXCR4) pathways; (2) neutrophil interaction through the unique SAA1-FPR2 pathway; (3) T cell modulation mediated by the exclusive CCL19-CCR7, MIF-(CD74+CD44), and MIF-(CD74+CXCR4) pathways; and (4) endothelial cell signaling through the specific CXCL3/CXCL6/CXCL8-ACKR1 pathways (**Figure 7H**). Additionally, we analyzed the biological pathways of DEGs between the positive and negative subpopulations of these two genes to uncover their associated biological pathways. KEGG enrichment analysis of the DEGs revealed upregulation of several inflammation-related signaling pathways, including NF-κB, IL-17, and TNF signaling pathways, in MME^+^ and TSPAN11^+^ GFs (**Supplementary Figures 3A, B**). In conclusion, these findings suggest that GFs expressing MME and TSPAN11 may possess a strong ability to interact with immune cells, potentially exacerbating immune responses and promoting the development of periodontitis.

### 3.9 The correlation between characteristic genes and immune cells

**Figure 8:**
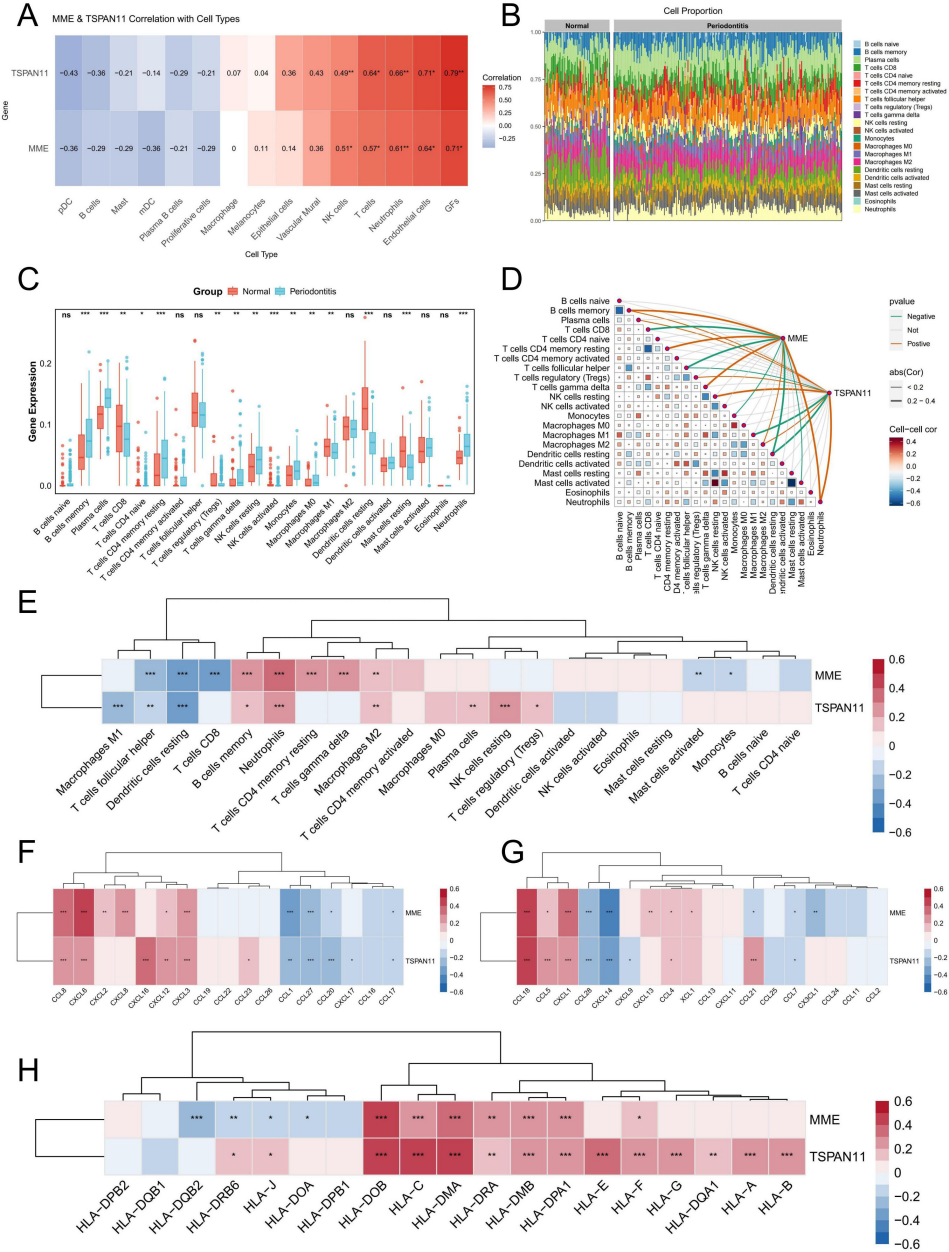
Immune cell infiltration analysis. **(A)** The association between MME and TSPAN11 and cell type was analyzed using scRNA-seq data. **(B)** A stacked bar plot depicting the relative abundance of immune cell types in normal and periodontitis groups. **(C)** Boxplot illustrating the proportion of 22 different kinds of immune cells in periodontitis versus normal samples. **(D)** The correlation between immune cells and their association with key characteristic genes. **(E)** A heatmap illustrating the correlation between MME, TSPAN11, and 22 types of infiltrating immune cells. **(F, G)** Heatmaps illustrating the correlation between chemokine-related genes and 2 feature genes. **(H)** A heatmap illustrating the correlation between HLA-related genes and 2 feature genes. *p < 0.05, **p < 0.01, ***p < 0.001. ns, no significance; GFs, Gingival fibroblasts.

Subsequently, we further used the scRNA-seq data and bulk RNA-seq data to investigate the correlation between these two characteristic genes and immune cells. The Spearman coefficient was used to evaluate the correlation of MME and TSPAN11 with various cell types at the single-cell level, and the results showed that MME and TSPAN11 were positively correlated with neutrophils, NK and T cells (**Figure 8A**). For bulk RNA-seq analysis, we first employed the CIBERSORT algorithm to explore and compare immune cell infiltration patterns between normal and periodontitis samples. The results revealed significant alterations in the proportions of multiple immune cell populations in both normal and periodontitis samples (**Figure 8B**). Specifically, periodontitis samples exhibited a marked upregulation in the infiltration levels of various immune cells, including neutrophils and plasma cells, which aligned with our scRNA-seq findings (**Figure 8C**). Subsequently, the relationship between immune cells and the identified PIGFs-related hub genes was investigated. The analysis revealed strong associations between these genes and specific immune cell populations. Specifically, MME showed a positive correlation with memory B cells and neutrophils, while TSPAN11 was positively correlated with Tregs, M2 macrophages, and neutrophils but negatively correlated with M1 macrophages (**Figures 8D, E**). Additionally, we explored the relationships between these genes and immune-related gene families, including HLA genes and chemokine-related genes, to elucidate their potential roles in immune regulation. The results indicated significant correlations between these genes and several chemokine-related genes and HLA genes (**Figures 8F-H**). In conclusion, MME and TSPAN11 were closely associated with diverse immune cells, chemokine-related genes and HLA genes, suggesting their potential role in mediating immune responses in the pathogenesis of periodontitis. These findings provided novel insights and potential therapeutic targets for the treatment of periodontitis.

### 3.10 Experimental verification

**Figure 9:**
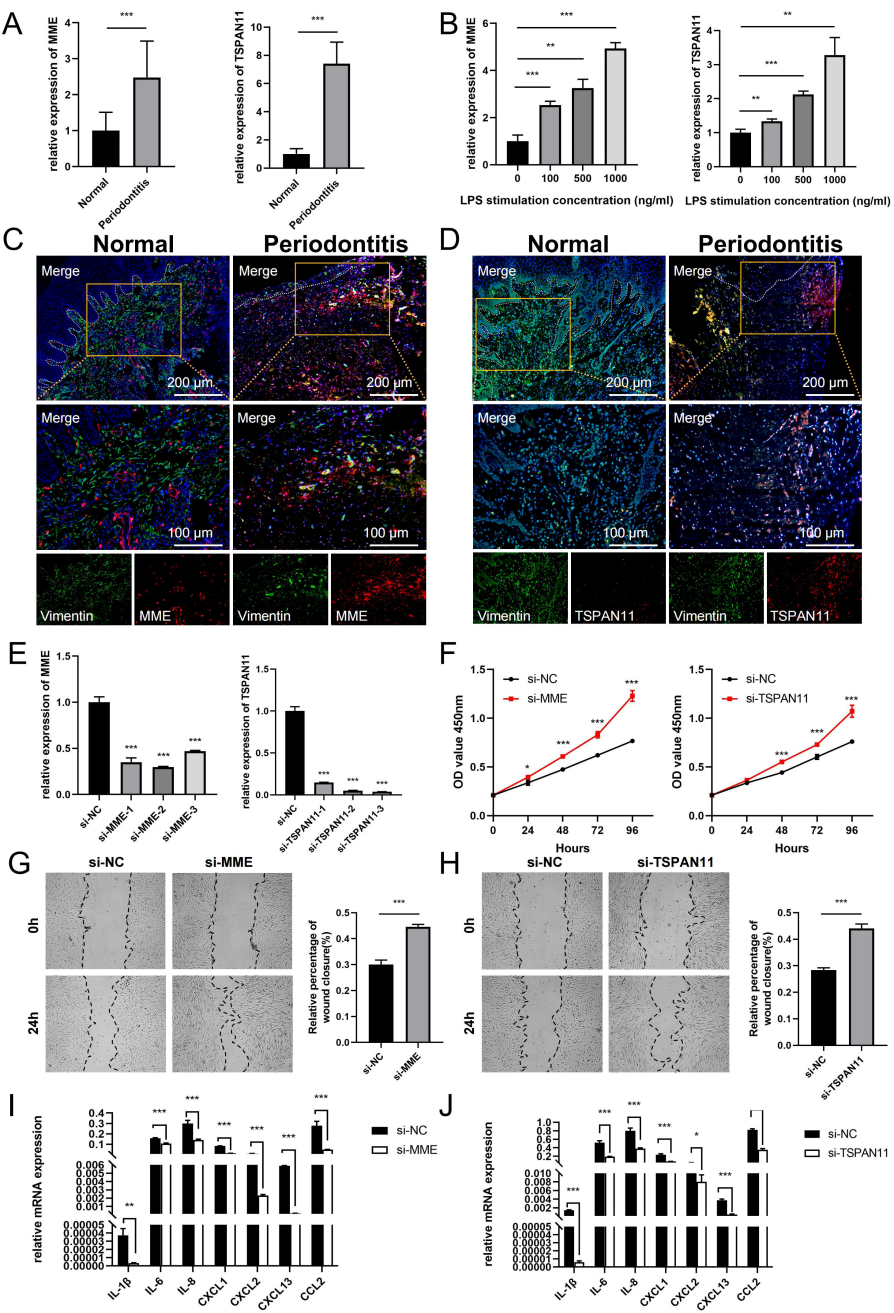
Expression and functional validation of characterized genes. **(A)** qRT-PCR results show the mRNA expression levels of MME and TSPAN11 in the gingivae of individuals in the healthy and periodontitis groups. GAPDH was used for normalization relative to the control group. **(B)** Expression levels of MME and TSPAN11 mRNA in GFs cultured with LPS concentration. **(C)** Immunofluorescence staining of MME, in which vimentin positive represents GFs. **(D)** Immunofluorescence staining of TSPAN11, in which vimentin positive represents GFs. **(E)** qRT-PCR analysis of MME and TSPAN11 expression in GFs cells treated with siRNAs. **(F)** The proliferation capacity of GFs transfected with NC, si-MME and si-TSPAN11 was detected by CCK-8 assay. **(G)** The migration and invasion ability of the cells treated with si-NC and si-MME were detected by wound healing. **(H)** The migration and invasion ability of the cells treated with si-NC and si-TSPAN11 were detected by wound healing. **(I)** The expression of various pro-inflammatory cytokines and chemokines in GFs transfected with si-NC and si-MME. **(J)** The expression of various pro-inflammatory cytokines and chemokines in GFs transfected with si-NC and si-TSPAN11. Data were shown as mean ± SD, *p < 0.05, **p < 0.01, ***p < 0.001. GFs, Gingival fibroblasts.

The bioinformatics analysis in this study indicated that MME and TSPAN11 expression was upregulated in periodontitis samples. To validate these findings, we performed qPCR to compare their expression levels between healthy and inflamed gingival tissues. Consistently, the qPCR results confirmed a significant upregulation of MME and TSPAN11 in inflamed gingival samples compared to healthy controls (**Figure 9A**). Meanwhile, we cultured GFs with LPS *in vitro* to simulate the inflammatory environment of periodontitis and evaluate the expression changes of these genes. As expected, qPCR analysis revealed a dose-dependent increase in MME and TSPAN11 expression with rising LPS concentrations (**Figure 9B**). Furthermore, immunofluorescence staining was performed to determine the localization of MME and TSPAN11 within gingival tissue. The results showed that both proteins were predominantly expressed in GFs, with higher levels observed in periodontitis tissues compared to healthy controls. These findings suggest that MME and TSPAN11 may play a functional role in GFs during the progression of periodontitis (**Figures 9C, D**).

Additionally, we investigated the function of MME and TSPAN11 in gingival fibroblasts (GFs). qRT-PCR results showed that the expression of MME and TSPAN11 was significantly downregulated following treatment with siRNA fragments. Among the three siRNAs used for knockdown, si-MME-2 and si-TSPAN11-3 were selected for further studies due to their higher silencing efficiency in GFs (**Figure 9E**). CCK-8 assay results revealed that downregulation of MME and TSPAN11 significantly promoted the proliferative activity of GFs (**Figure 9F**). Moreover, wound healing assays demonstrated that silencing MME and TSPAN11 enhanced the invasion and migration abilities of GFs (**Figures 9G, H**). Given that KEGG pathway analysis indicated significant elevation of multiple inflammatory pathways in MME^+^ GFs and TSPAN11^+^ GFs, we further examined the effect of MME and TSPAN11 on downstream inflammatory cytokines and chemokines. Our findings revealed that downregulation of MME and TSPAN11 markedly reduced the expression of IL-1β, IL-6, IL-8, CXCL1, CXCL2, CXCL13, and CCL2 in GFs treated with LPS (**Figures 9I, J**). Collectively, these results strongly suggest that MME and TSPAN11 play critical roles in regulating GFs’ inflammatory responses and cellular behaviors, highlighting their potential as therapeutic targets for periodontitis.

## 4 Discussion

With the advent of scRNA-seq technology, single-cell expression profiling has enabled the exploration of disease pathogenesis at the cellular level, overcoming the information loss inherent in conventional transcriptomic sequencing and offering novel insights into the identification of potential disease-associated genes. In this study, we integrated scRNA-seq and bulk RNA-seq analyses to investigate the underlying mechanisms of periodontitis pathogenesis. After processing and analyzing the scRNA-seq data, we a distinct subpopulation of GFs that was strongly associated with inflammatory responses, and their proportion in periodontitis was greater than that of healthy controls. Analysis of its cellular interactions showed that PIGFs showed stronger cellular communication ability compared with other GFs subtypes, indicating its importance in the pathogenesis of periodontitis. To validate our findings, we analyzed another periodontitis-related scRNA-seq dataset (GSE171213), further reinforcing the robustness and reliability of our results (**Supplementary Figures 4A-L**). Additionally, to identify characteristic genes closely associated with this subpopulation, we integrated the uniquely highly expressed genes within this subset with DEGs and modular core genes from bulk RNA-seq data, ultimately identifying a total of 27 candidate genes. LASSO regression and RF, two machine learning algorithms, were finally utilized to determine the two best signature genes (MME and TSPAN11) that are highly related with the onset or progression of periodontitis. ROC analysis suggested their potential relevance in disease risk assessment. Through immunoinfiltration analysis, we discovered that they were strongly connected to a range of immune cells, HLA-related genes, and chemokine-related genes, indicating a close relationship between these characteristic genes and immunity. Finally, we explored the functions of these two genes through a variety of experimental techniques and found that inhibition of these two genes can promote the proliferation and migration of GFs and inhibit the production of inflammatory factors in GFs. In conclusion, our study identifies PIGFs associated with periodontitis and reveals their role in the pathogenesis of periodontitis. MME and TSPAN11, two characteristic genes associated with PIGFs, may become targets for the treatment of periodontitis in the future.

Previous studies have shown that GFs (6, 8, 16) and epithelial cells (6, 17), the primary cellular components of gingival mucosal tissue, contribute to the inflammatory phase of periodontitis by secreting chemokines such as CXCL1, CXCL2, CXCL12, CCL2, and CCL19, which play a crucial role in recruiting lymphocytes and neutrophils to the affected gingival tissue and amplifying the inflammatory response (18). Moreover, GFs overproduce additional inflammatory mediators, such as prostaglandin E2 (PGE2), MMPs (MMP1, MMP3, MMP8, and MMP9), and cytokines (IL-1β and IL-6) (19, 20). In this study, we found that PIGFs have a strong ability to output signals by “cellchat” analysis. It can participate in the occurrence of periodontitis by releasing a variety of chemokines to interact with other cells. Specifically, PIGFs may interact with NK/T cells, mDC, and B cells through CXCL12-CXCR4 axis, which may recruit multiple immune cells to the site of inflammation thereby destroying the integrity of periodontal tissue. In addition, it can act on endothelial cells via the CXCL1/ACKR1 and CXCL13/ACKR1 axes, and we hypothesized that this may promote endothelial cell angiogenesis, leading to gingival bleeding in periodontitis. The above results indicated the important regulatory role of PIGFs in the periodontal inflammatory process.

By integrating the PIGF signature gene with WGCNA and various machine learning approaches, we identified two key signature genes associated with PIGFs that may play a significant role in the progression of periodontitis. The MME gene is located on human chromosome 3q21-27, which functions as a tumor suppressor and is linked to a number of malignancies (21). It can also regulate inflammatory responses and insulin signaling in white preadipocytes (22). Furthermore, it encodes for a neutral endopeptidase, the expression of which is associated with the severity of the periodontitis and is mostly expressed on neutrophils and GFs associated with periodontitis (23). There are few studies on TSPAN11. The orientation of bone stroma tissue is reported to be determined by TSPAN11-mediated fibrous adhesion patch assembly. TSPAN11 silencing significantly disrupts the arrangement of osteoblasts, and the arrangement is orthogonal as the bone matrix is further constructed (24). This study revealed that MME and TSPAN11 are primarily expressed in fibroblasts, and the results of immunological correlation and cell communication suggest that they are intimately associated with several immune cells. Functionally, inhibition of these two genes in fibroblasts resulted in downregulation of several inflammatory cytokines and chemokines, which is consistent with bioinformatics results, suggesting that these two genes may be targets for the treatment of periodontitis.

The research has certain shortcomings. First, our research mainly used publicly accessible scRNA-seq and bulk RNA-seq data, which lacked essential clinical details such as the severity, stage, or grading of periodontitis. Consequently, the absence of comprehensive clinical context may limit our ability to fully explore the role of PIGFs in periodontitis across different disease stages or severity levels. Future studies incorporating well-annotated clinical data would help to further elucidate the functional contributions of PIGFs in periodontitis progression. Second, although we used the “Cell Chat” package in R to explore the interactions of PIGFs with other cells in the pathogenesis of periodontitis, these results require further validation. Additionally, *in vitro* experiments suggest that PIGFs-related hub genes may contribute to the development of periodontitis by mediating the release of inflammatory factors from GFs. However, the specific mechanism of its action still needs further study. Finally, we used immunofluorescence staining and qPCR to confirm the outcomes of the bioinformatics analysis. Nevertheless, considering the specificity of patient samples and the limited applicability of gingival tissue excision in clinical diagnostics, further validation using non-invasive approaches, such as saliva and gingival crevicular fluid, and large-scale clinical cohorts is needed to assess the broader relevance of these feature genes.

## 5 Conclusion

By scRNA-seq analysis, we identified a subpopulation of fibroblasts closely related to the inflammatory response, which may interactively participate in the pathogenesis of periodontitis by exacerbating the immune response. Additionally, we identified two genes (MME and TSPAN11) associated with this subpopulation that may be involved in the pathogenesis of periodontitis by promoting immune cell recruitment and exacerbating the immune response. Functionally, inhibiting these two genes can promote the proliferation and migration of GFs and reduce the production of chemokines and inflammatory factors in GFs, thereby mitigating the progression of periodontitis. Our study may provide novel insights into the pathophysiology of periodontitis, highlighting the critical role of GFs subpopulations and their associated genes in disease development, which may provide hope for more effective treatments for periodontitis.

## Data Availability

Publicly available datasets were analyzed in this study. This data can be found at GEO data repository (https://www.ncbi.nlm.nih.gov/geo/) and includes the accession numbers: GSE164241, GSE16134, and GSE10334.
