# Supplementary material for: Exploration the role of pro-inflammatory fibroblasts and related markers in periodontitis: combing with scRNA-seq and bulk-seq data
Source: Front Immunol. 2025 Apr 30;16:1537046. doi: 10.3389/fimmu.2025.1537046 (PMC12074970; doi:10.3389/fimmu.2025.1537046)
Supplement: Supplementary file 1 [file SupplementaryFile1.docx]

Supplementary Material


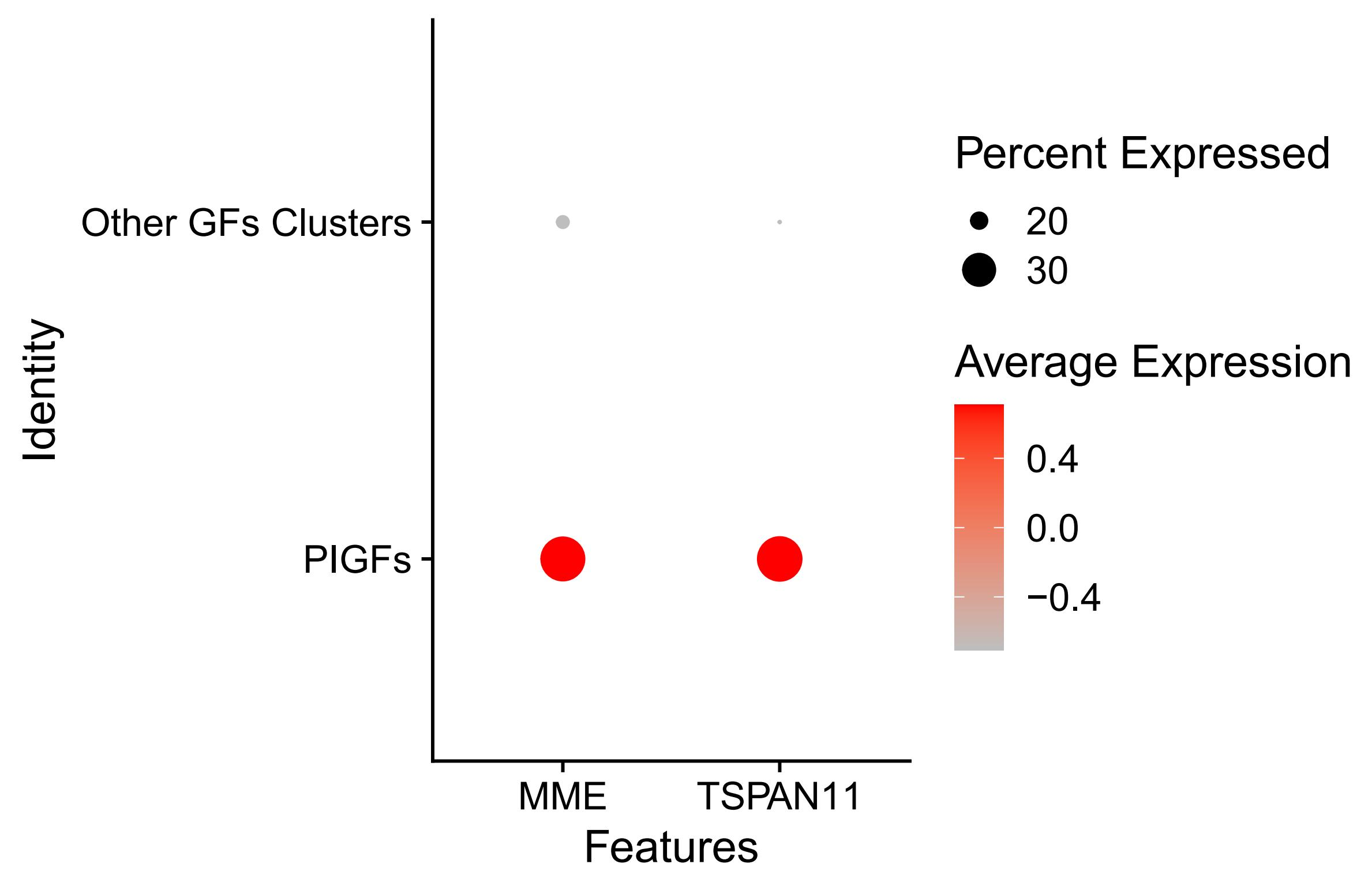


B


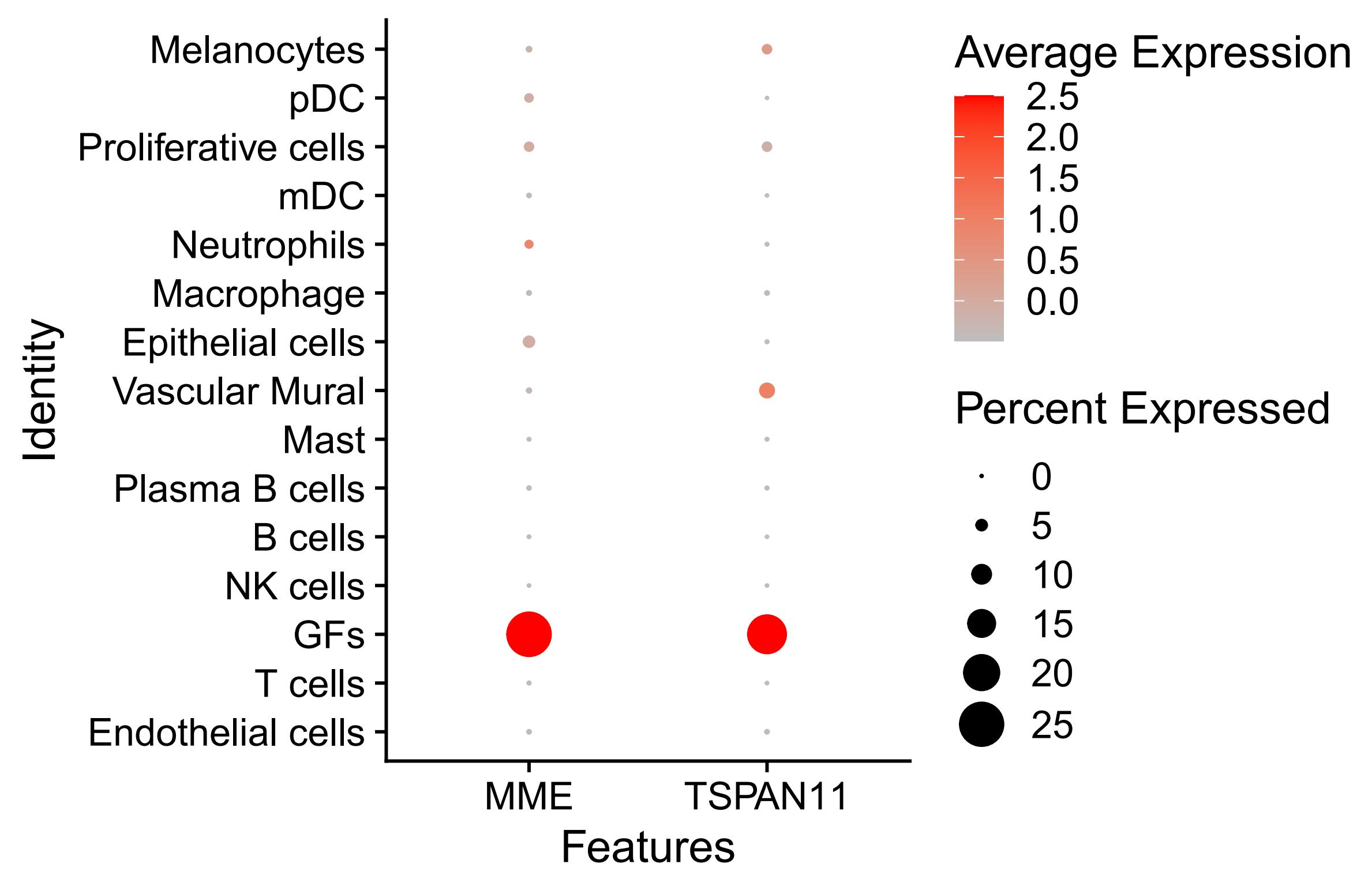


A

**Supplementary Figure 1.** (A) Expression of MME and TSPAN11 in 15 different cell clusters. (B) Expression of MME and TSPAN11 in the GFs subpopulation. GFs: Gingival fibroblasts.
