# Supplementary material for: Exploration the role of pro-inflammatory fibroblasts and related markers in periodontitis: combing with scRNA-seq and bulk-seq data
Source: Front Immunol. 2025 Apr 30;16:1537046. doi: 10.3389/fimmu.2025.1537046 (PMC12074970; doi:10.3389/fimmu.2025.1537046)
Supplement: Supplementary file 2 [file SupplementaryFile2.docx]

Supplementary Material


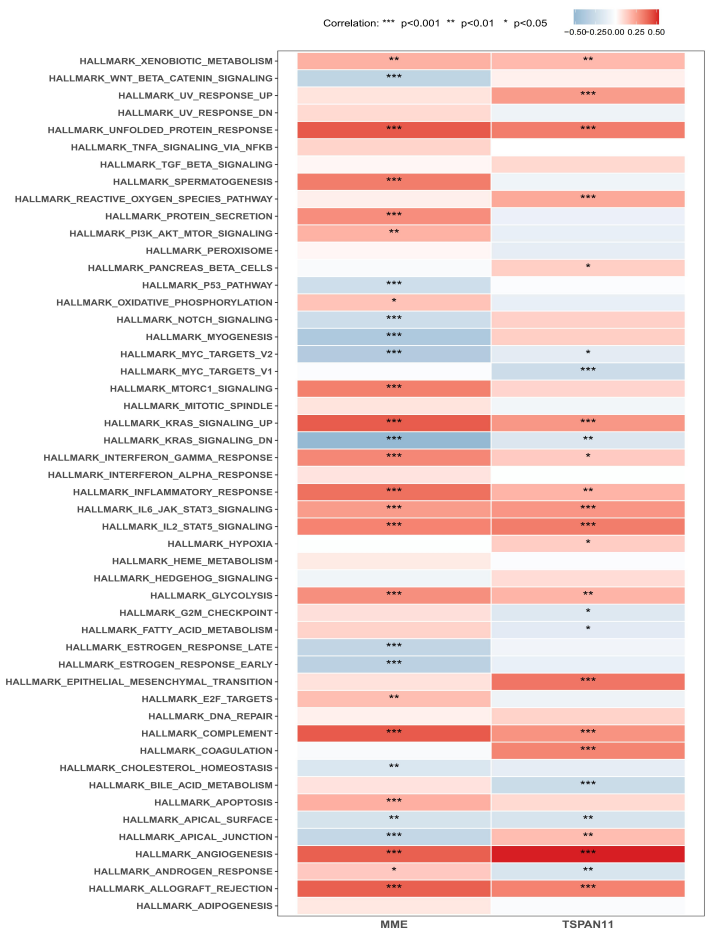


B


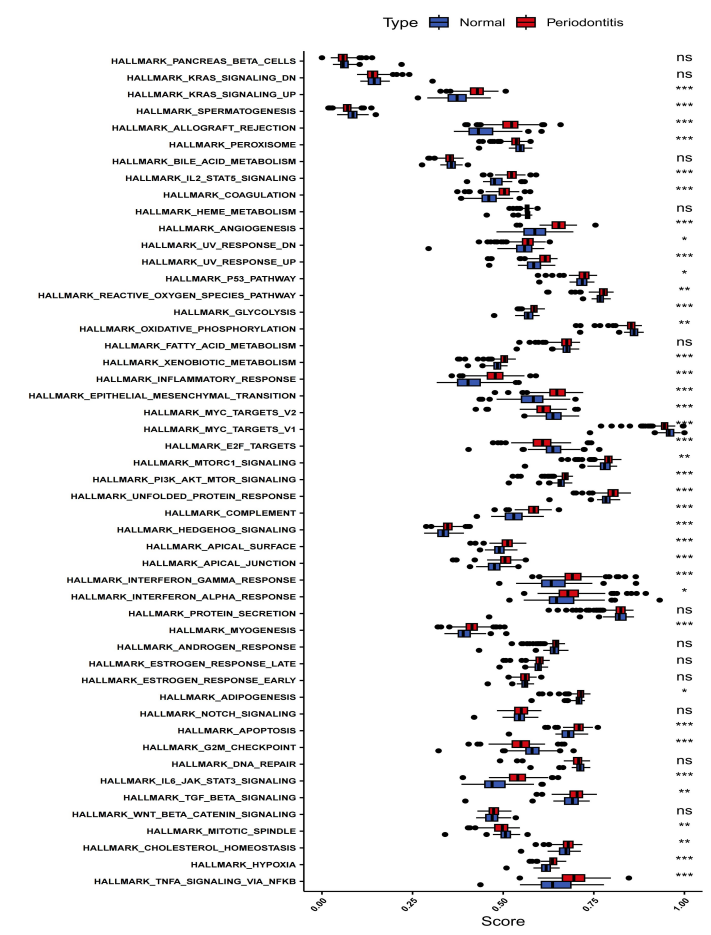


A

**Supplementary Figure 2.** (A) The specific distribution of 50 hallmark gene sets in the periodontitis group and control group samples. (B) The correlation analysis of 50 hallmark gene sets and 2 feature genes.
