# Supplementary material for: Exploration the role of pro-inflammatory fibroblasts and related markers in periodontitis: combing with scRNA-seq and bulk-seq data
Source: Front Immunol. 2025 Apr 30;16:1537046. doi: 10.3389/fimmu.2025.1537046 (PMC12074970; doi:10.3389/fimmu.2025.1537046)
Supplement: Supplementary file 3 [file SupplementaryFile3.docx]

Supplementary Material

B


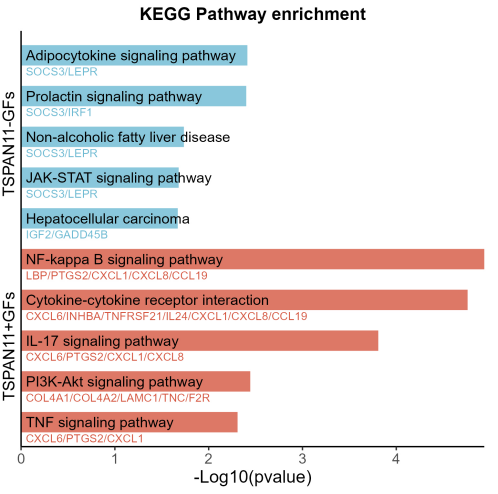


A


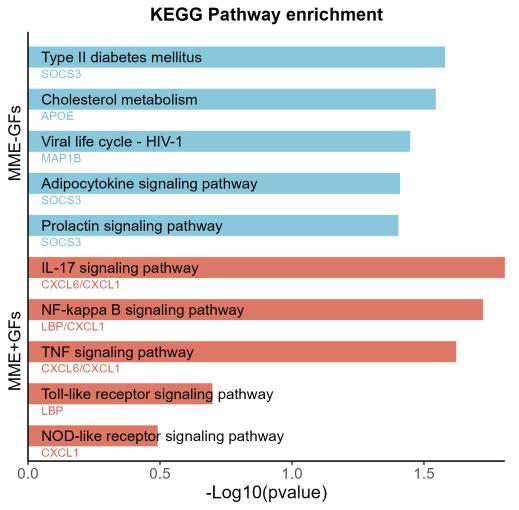


**Supplementary Figure 3.** (A-B) KEGG analysis revealed differential pathways between MME-positive/negative and TSPAN11-positive/negative subpopulations in GFs. GFs, Gingival fibroblasts.
