# Supplementary material for: Exploration the role of pro-inflammatory fibroblasts and related markers in periodontitis: combing with scRNA-seq and bulk-seq data
Source: Front Immunol. 2025 Apr 30;16:1537046. doi: 10.3389/fimmu.2025.1537046 (PMC12074970; doi:10.3389/fimmu.2025.1537046)
Supplement: Supplementary file 4 [file SupplementaryFile4.docx]

Supplementary Material

**Supplementary methods**: In this study, we additionally analyzed another scRNA-seq dataset (GSE171213) derived from human chronic periodontitis and clinically healthy periodontal tissues. The dataset included gingival tissue samples from 4 healthy individuals and 5 patients with periodontitis. Following the same analytical pipeline as in our original study, we performed quality control, normalization, data integration, batch effect correction, principal component analysis (PCA), cell clustering, and t-SNE-based dimensionality reduction using the “Seurat” package in R. Subsequently, the gingival fibroblasts (GFs) subpopulation was further subclustered, and its interactions with other cell types were systematically examined using the “CellChat” software package.


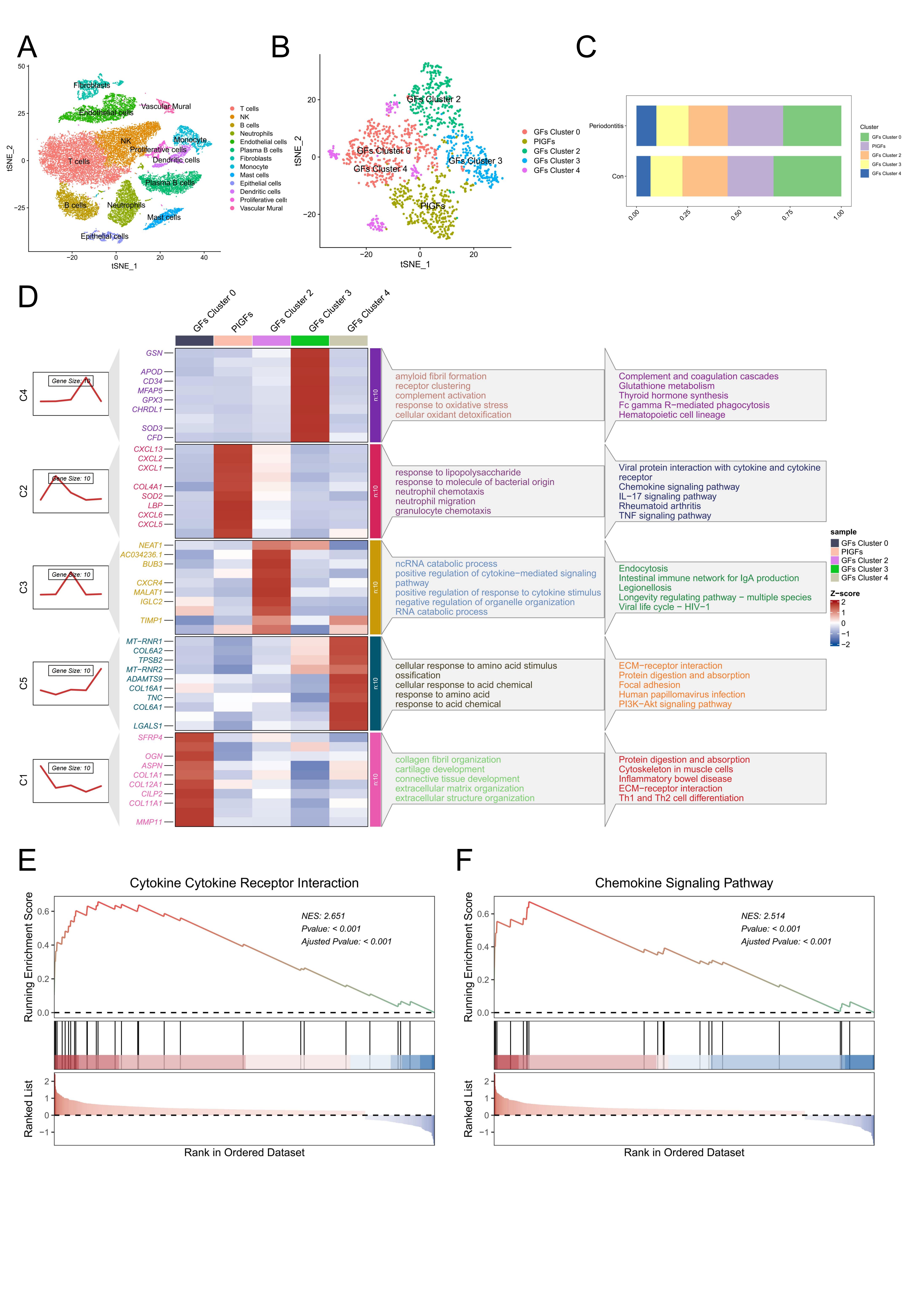


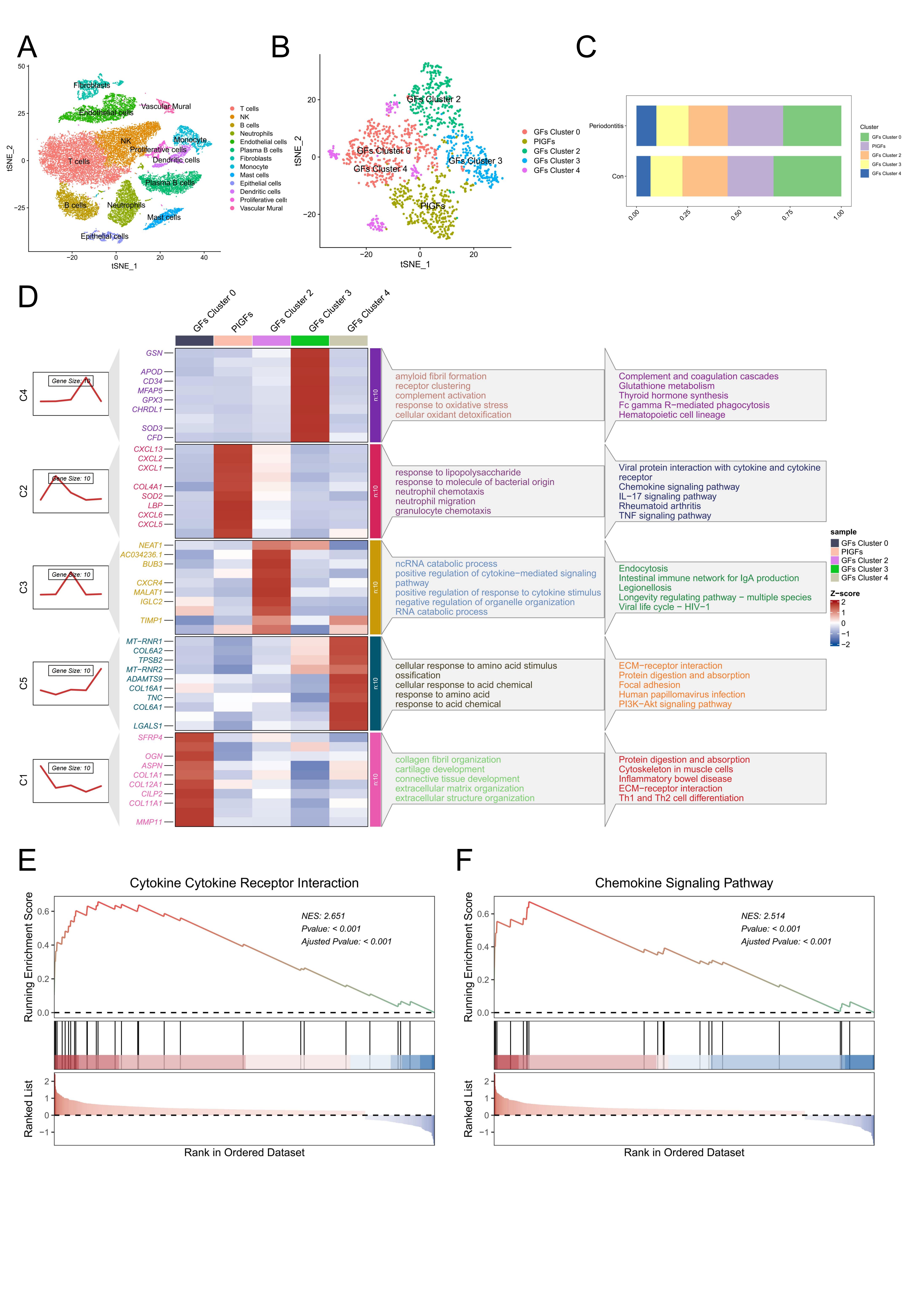


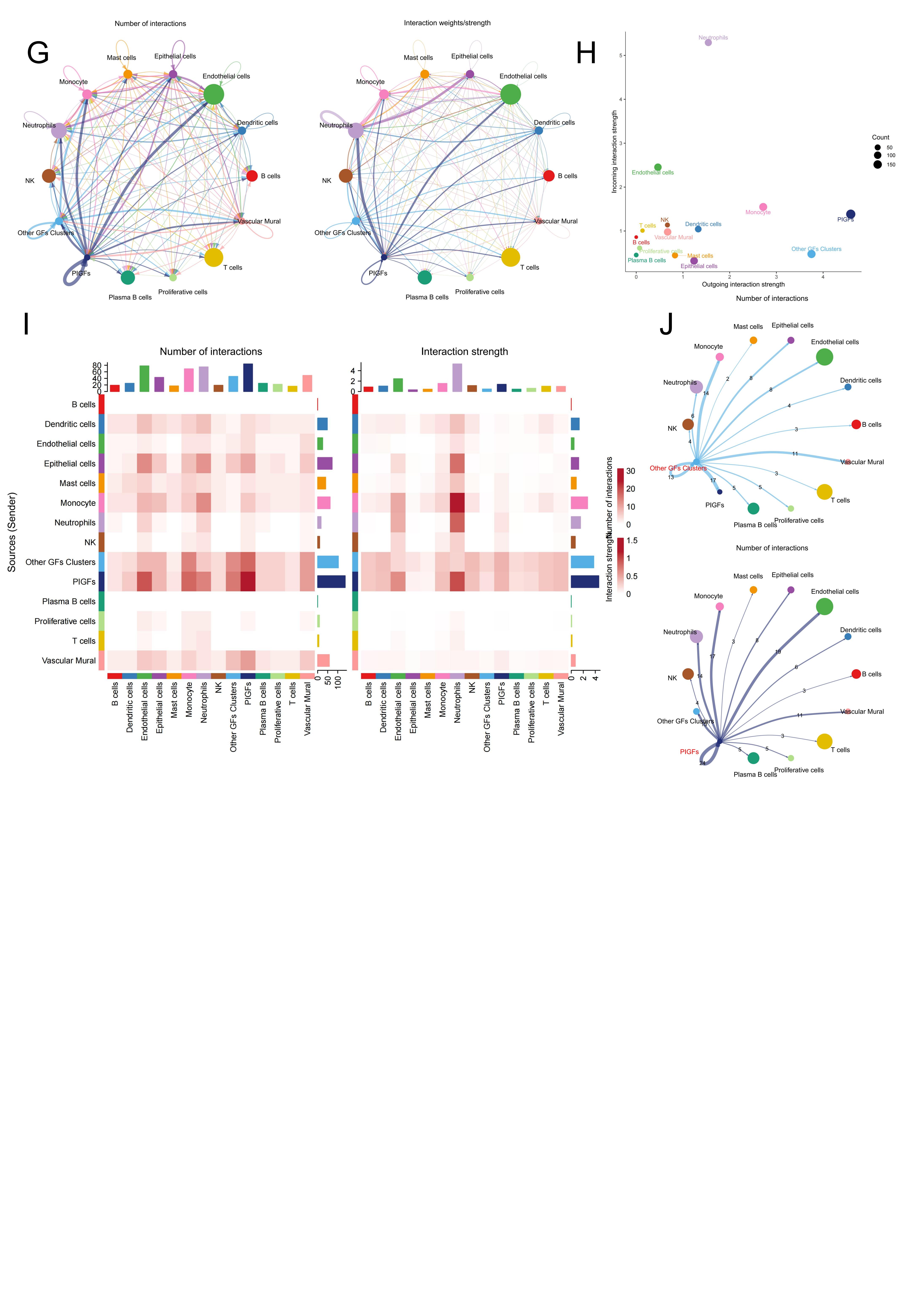


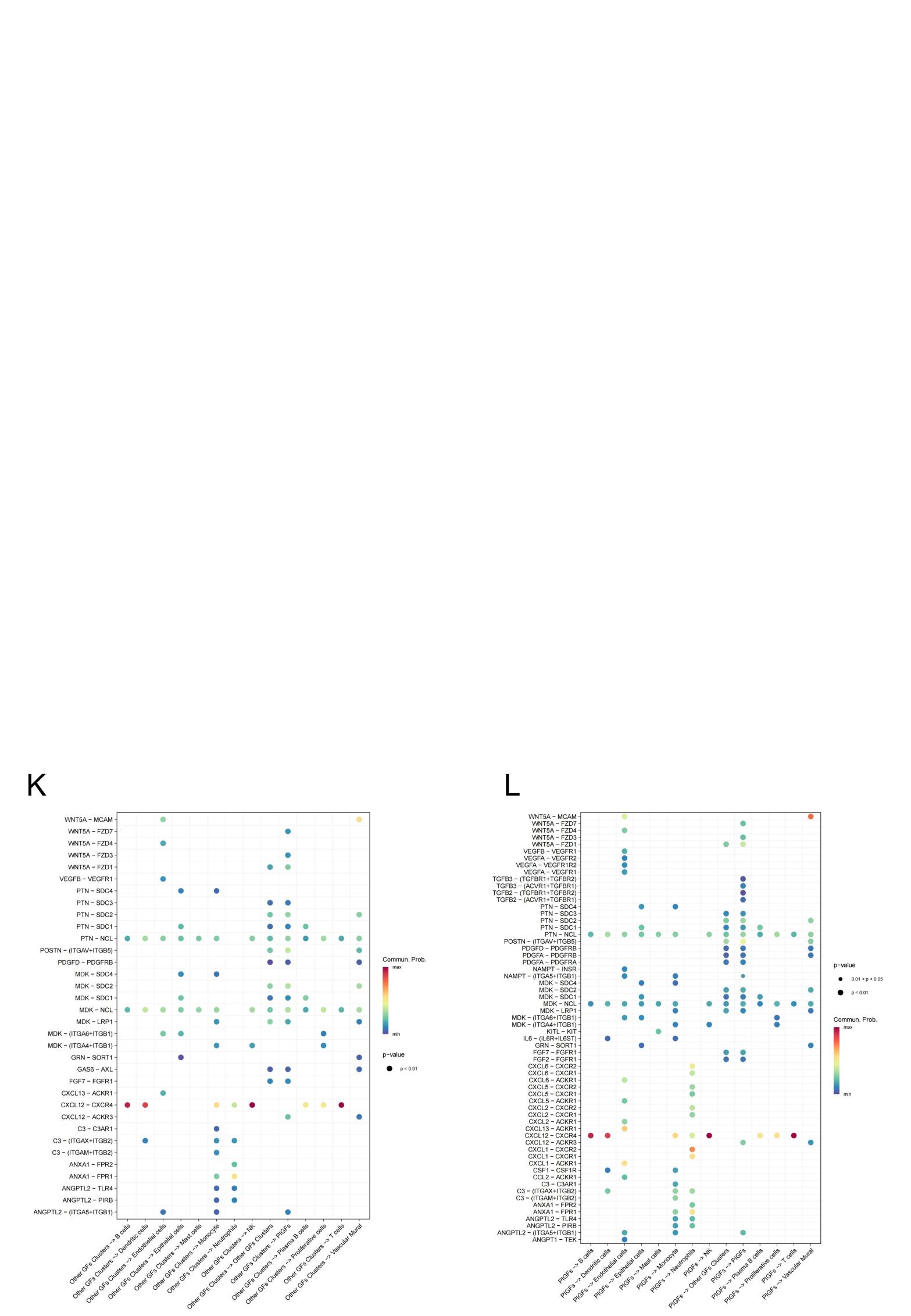


**Supplementary Figure 4**. The identification of PIGFs in GSE171213 using scRNA-seq analysis. (A) The t-SNE plot visualizes the distribution of 13 cell types in gingival samples. (B) Subclustering of GFs in normal and periodontitis samples further identified 5 distinct subtypes. (C) Cell proportions of GFs subclusters in the gingival tissues of normal and periodontitis patients. (D) Heatmap showing representative differentially expressed genes between each celltype. The left panel depicts the dynamic expression patterns of representative DEGs across each cell type. The right panel presents the corresponding biological functions and pathways associated with each cell type, as identified through GO and KEGG analysis. (E-F) GSEA enrichment plots for representative signaling pathways upregulated in PIGFs compared to other GFs. (G) Circle plots depict the number and strength of ligand-receptor interactions between pairs of cell populations. (H) A scatter plot reveals the variations in incoming and outgoing interaction strengths across all cell types. (I) Heatmaps illustrate the quantity and intensity of interactions between the PIGFs and other GFs subpopulations with other cell types. (J) Circle plots depict the number and intensity of interactions between other GFs and PIGFs and other cells, respectively. The edges indicated the strength of the interactions, with thicker edges indicating stronger interactions. Numbers on the edges indicated the number of communication signals between the two cell types. (K-L) Bubble plot showing the significant ligand-receptor pairs between other GFs and PIGFs and other cells, respectively. The size of each dot represents the significance level (p-value), with larger dots indicating greater statistical significance. The color gradient reflects the strength of cellular communication, where redder hues denote stronger interactions. GFs, Gingival fibroblasts; PIGFs, Pro-inflammatory gingival fibroblasts.
