# Supplementary material for: Exploration the role of pro-inflammatory fibroblasts and related markers in periodontitis: combing with scRNA-seq and bulk-seq data
Source: Front Immunol. 2025 Apr 30;16:1537046. doi: 10.3389/fimmu.2025.1537046 (PMC12074970; doi:10.3389/fimmu.2025.1537046)
Supplement: Supplementary file 5 [file Table1.docx]

Supplementary Material

| Gene | Forward 5’-3’ | Reverse 5’-3’ |
| --- | --- | --- |
| GAPDH | CTTTGGTATCGTGGAAGGACTC | GTAGAGGCAGGGATGATGTTCT |
| MME | TGTAAAGCCACCCACAAACA | GTTGCTGCCTGTTGACTTGA |
| TSPAN11 | CATCTTTGCGGGCGTACTTG | CAGGCAGAAATACGTGGAGAG |
| IL-1β | AGAAGTACCTGAGCTCGCCA | CTGGAAGGAGCACTTCATCTGT |
| IL-6 | AGGCACTGGCAGAAAACAAC | TTTTCACCAGGCAAGTCTCC |
| IL-8 | GTGTGAAGGTGCAGTTTTGC | TGTGGTCCACTCTCAATCACTC |
| CXCL1 | AGTCATAGCCACACTCAAGAATGG | GATGCAGGATTGAGGCAAGC |
| CXCL2 | CAAACCGAAGTCATAGCC | GAACAGCCACCAATAAGC |
| CXCL13 | GCTTGAGGTGTAGATGTGTCC | CCCACGGGGCAAGATTTGAA |
| CCL2 | AGAATCACCAGCAGCAAGTGTCC | TCCTGAACCCACTTCTGCTTGG |

**Supplementary Table 1.** Sequences of the primers used for RT-qPCR.
